# G Protein Coupled Receptor Kinase 3 Regulates Breast Cancer Migration, Invasion, and Metastasis

**DOI:** 10.1371/journal.pone.0152856

**Published:** 2016-04-06

**Authors:** Matthew J. Billard, David J. Fitzhugh, Joel S. Parker, Jaime M. Brozowski, Marcus W. McGinnis, Roman G. Timoshchenko, D. Stephen Serafin, Ruth Lininger, Nancy Klauber-Demore, Gary Sahagian, Young K. Truong, Maria F. Sassano, Jonathan S. Serody, Teresa K. Tarrant

**Affiliations:** 1 Thurston Arthritis Research Center and the Department of Medicine, Division of Rheumatology, Allergy, and Immunology, University of North Carolina, Chapel Hill, NC 27599, United States of America; 2 Department of Genetics, University of North Carolina at Chapel Hill, Chapel Hill, NC, 27599, United States of America; 3 Lineberger Comprehensive Cancer Center, University of North Carolina Chapel Hill, NC 27599, United States of America; 4 Department of Microbiology and Immunology, University of North Carolina, Chapel Hill, NC, 27599, United States of America; 5 Department of Pathology and Laboratory Medicine, University of North Carolina, Chapel Hill, NC 27599, United States of America; 6 Department of Surgery, Division of Surgical Oncology, University of North Carolina, Chapel Hill, NC 27599, United States of America; 7 Department of Medicine, Division of Hematology Oncology, University of North Carolina, Chapel Hill NC, 27599, United States of America; 8 Department of Developmental, Molecular & Chemical Biology, Tufts University, Medford, MA 02155, United States of America; 9 Department of Biostatistics, Gillings School of Global Public Health, University of North Carolina, Chapel Hill, NC 27599, United States of America; 10 Department of Pharmacology, University of North Carolina, Chapel Hill, NC 27599, United States of America; University of Florida, UNITED STATES

## Abstract

Triple negative breast cancer (TNBC) is a heterogeneous disease that has a poor prognosis and limited treatment options. Chemokine receptor interactions are important modulators of breast cancer metastasis; however, it is now recognized that quantitative surface expression of one important chemokine receptor, CXCR4, may not directly correlate with metastasis and that its functional activity in breast cancer may better inform tumor pathogenicity. G protein coupled receptor kinase 3 (GRK3) is a negative regulator of CXCR4 activity, and we show that GRK expression correlates with tumorigenicity, molecular subtype, and metastatic potential in human tumor microarray analysis. Using established human breast cancer cell lines and an immunocompetent *in vivo* mouse model, we further demonstrate that alterations in *GRK3* expression levels in tumor cells directly affect migration and invasion *in vitro* and the establishment of distant metastasis *in vivo*. The effects of GRK3 modulation appear to be specific to chemokine-mediated migration behaviors without influencing tumor cell proliferation or survival. These data demonstrate that GRK3 dysregulation may play an important part in TNBC metastasis.

## 1. Introduction

Breast cancer is the most prevalent and deadliest form of cancer in women world-wide and triple negative breast cancers (TNBC) account for approximately 15–20% of all breast cancers [1, 2]. Triple negative breast cancer is a heterogeneous disease defined by negative expression for estrogen receptor (ER) and progesterone receptor (PR), and normal (low) expression levels of Her2. Recent molecular characterization has identified five breast cancer subtypes (luminal A, luminal B, HER2-enriched, basal-like, and claudin-low). Approximately 90% of TNBC tumors are basal-like or claudin-low subtypes, with 70% of basal-like being identified as triple-negative and 80% of claudin-low identified as ER-/-, PR-/-, Her2-/-[3]. Triple negative breast cancers are often highly invasive, metastatic, possess a high risk for relapse, and carry a poor prognosis [4, 5], and the different genetic subtypes can be associated with organ-specific metastasis and relapse [6]. As hormone receptor antagonism is less applicable in TNBC, alternative targets are being investigated to better understand pathogenesis and to design therapeutics for these patients.

The G protein coupled receptors (GPCR), such as CXCR4, and its chemokine ligand CXCL12, have been identified as important regulators of metastasis and tumor behavior in breast cancer [7–9]. CXCL12 is highly expressed at common sites of breast cancer metastasis, such as the lymph nodes, liver, bone marrow, and lungs [7], and CXCR4 on tumor cells is critical for growth and migration [9]. While CXCR4 expression on tumor cells is important for metastasis, work published by Holland, et al. has emphasized that functional activation and signaling of CXCR4 in breast cancer cells may be more predictive of malignant potential than quantitative surface expression [10]. Thus, the intracellular signaling responses of chemokine receptor activation are of critical importance to metastasis and the malignant phenotype.

G protein coupled receptor kinases (GRKs) are signaling regulators of agonist-activated chemokine receptors. Agonist activation leads to phosphorylation of the receptor tail by GRK, the recruitment of beta-arrestin, uncoupling of heterotrimeric G proteins, and ultimately desensitization and internalization of the receptor [11, 12]. Thus, GRKs serve as negative regulators of GPCRs. Reduced expression of GRK3, 4, and 6 have previously been reported to increase tumor malignancy of glioblastoma, ovarian tumors, and medulloblastoma, respectively, through dysregulation of GPCR signaling [13–15]. In the glioblastoma model specifically, decreased GRK3 expression resulted in abnormally sustained CXCR4 signaling and enhanced tumor growth [14]. Further, GRK3 has been shown to regulate CXCL12-mediated cellular migration and CXCR4 internalization and MAPK signaling in human and mouse models of immune deficiency and inflammation [16, 17].

Given the aforementioned importance of GRK3 in CXCR4 signaling regulation and the role of CXCL12 and CXCR4 in malignancy [9, 18, 19], we hypothesized that GRK3 regulation of CXCL12/CXCR4, as well as potentially other chemokine receptor interactions, may further distinguish differences in metastatic potential and prognosis in patient subsets within TNBC. To predict the relevance of GRK3 in human breast cancer subtypes, we analyzed TCGA and publically available microarray data for GRK correlations with molecular subset distinction and metastasis. To test the potential mechanism of GRK3 regulation of CXCR4 in disease, human TNBC cell lines MDA-MB-231 (highly invasive) and MDA-MB-468 (weakly invasive) [20] were examined for *GRK3* and *CXCR4* expression and for functional migration phenotypes when *GRK3* expression was altered. To test the effects of GRK3 on tumor growth and metastasis *in vivo*, we used GRK3-deficient mammary tumor cells derived from Balb/c (66cl4-luc) as a representative TNBC model system in an immunocompetent mouse [21]. Our studies show that *GRK3* is decreased in specific molecular subsets of TNBC with increased metastasis in humans and that GRK3 expression can predict the functional phenotype of migration and invasion in both *in vitro* and *in vivo* model systems. Therefore, defining the relative expression of *GRK3* in relationship to chemokine receptors like *CXCR4* in TNBC may provide prognostic information for the aggressiveness of tumors and help discriminate patient subsets with the greatest potential for metastasis beyond their chemokine receptor surface expression alone.

## 2. Materials and Methods

### 2.1. Human breast cancer genetic analysis

The TCGA database was analyzed, as well as public microarray datasets, as previously described [6]. Specifically, these microarrays sets are deposited in the Gene Expression Omnibus with the accession numbers GSE26338, GSE2034, GSE12276, and GSE2603. Dataset NKI295 was published prior to the adoption of GEO and can be found separately (http://ccb.nki.nl/data/). The data was combined using Distance Weighted Discrimination [22] to remove the systematic biases of different microarray sets and standardized to zero mean and unit variances prior to other analyses. Samples in the normalized data were assigned to the five subtypes (luminal A, luminal B, Her2-enriched, basal-like, and normal) using the PAM50 classifier [23]. Assignment of claudin-low was performed according to the protocol described in Prat et al.[3] Testing for differential expression of the candidate genes associated with subtype was performed using ANOVA. Survival analysis was performed by grouping expression levels into tertiles, then testing for association with metastasis free survival using the log rank test and visualized with Kaplan Meier plots.

### 2.2. Mice and cell lines

*In vivo* experiments were performed in Balb/c mice. All animals were cared for under standard Institutional Animal Care and Use Committee (IACUC)-approved protocols in the Association for Assessment and Accreditation of Laboratory Animal Care-accredited vivarium at the University of North Carolina. Luciferase-tagged murine breast cancer line 66cl4-luc (generated by Dr. G. Sahagian) [24] and human breast cancer lines MDA-MB-468, MDA-MB-453, MDA-MB-231, DU4475, MCF-7, BT-474, SK-BR3, Hs578T, and ZR-75-1 were obtained from the University of North Carolina Lineberger Cancer Center Tissue Culture Facility (TCF) and maintained according to ATCC culture recommendations. Cell lines with stable lentiviral-mediated expression of GRK3-knockdown shRNA or plasmid-mediated overexpression constructs were cultured in medium with the addition of selection antibiotics puromycin and/or gentamicin at concentrations determined empirically by kill curve assays.

### 2.3. Lentiviral shRNA-mediated GRK3 silencing

Five target shRNAs were obtained for both human and mouse GRK3, which were cloned into pLKO.1.-CMV (Sigma, St. Louis, MO). Target and non-target vectors were packaged into lentiviral particles by Sigma or by the UNC Lenti-shRNA Core Facility. Each of these was transduced into the appropriate human (MDA-MB-468) or mouse (66cl4-luc) breast cancer lines and selected by antibiotic resistance. These clones were screened via quantitative real-time PCR (qRT-PCR) for *GRK3* expression and confirmed with immunoprecipitation protein blotting (S1 and S2 Figs). CXCR4 surface protein expression was also tested by flow cytometry and confirmed to be unaffected (S3 Fig). The target sequence producing the greatest GRK3-specific knockdown with the fewest off-target effects (judged by lack of effect on housekeeping genes IDUA, 18S, CXCR4, CXCR7 and GRK2—which has the closest sequence homology to GRK3) was selected for use in further assays: human ADRBK2, TRCN0000002034, sequence 5'-CCGG-CAGTAAATGCAGACACAGATA-CTCGAG-TATCTGTGTCTGCATTTACTG-TTTTT-3'; mouse ADRBK2, TRCN0000022703, sequence, 5’-CCGG-GCAGCATGTGTACTTACGGAA-CTCGAG-TTCCGTAAGTACACATGCTGC-TTTTT-3’.

### 2.4. Overexpression of GRK3

MDA-MB-231 cells were transiently transfected with GRK3 or empty control plasmid (pcDNA3.1 mCherry) using Lipofectamine LTX with Plus Reagent (Life Technologies, Grand Island, NY) per manufacturer instructions. Following 20 hours of culture with plasmid, cells were washed and used after 2-24hr rest in complete media or were serum-starved, where appropriate, prior to assay. Transient transfection efficiency was routinely 30–40% determined by microscopy for mCherry expression. GRK3 overexpression was confirmed by Western blot (S2 Fig).

### 2.5. Quantitative Real Time PCR

Cells were grown in appropriate culture media to approximately 75% confluence. Total RNA was prepared using a Qiagen RNeasy kit and cDNA synthesized with Superscript II reverse transcriptase (Invitrogen) followed by qRT-PCR. Fold differences in expression were calculated using the 2^-ΔCt^ method compared to housekeeping gene IDUA [17]. Transcript copy number was based on the standard curve method [25]. Human primers for qRT- PCR are listed as 5’to 3’ sequence as follows: Human GRK2 forward ACTTCAGCGTGCATCGCAT, GRK2 reverse GCTTTTTGTCCAGGCACTTCAT, GRK3 forward AAGCCTTCGAGGTGACATTTTT, GRK3 reverse GCAACCATAAACTTCCCCGAATC, IDUA forward CTCGGGCCACTTCACTGAC, IDUA reverse CAGTCCGTACCTACCGATGTAT, CXCR7 forward TGCATCTCTTCGACTACTCAGA, CXCR7 reverse GGCATGTTGGGACACATCAC, CXCR4 forward CTCACTGACGTTGGCAAAGA, and CXCR4 reverse AGGAAGCTGTTGGCTGAAAA.

### 2.6. CXCR4 internalization assay

According to previously published protocols [17], transiently transfected control and GRK3-overexpressed MDA-MB-231 cells were treated for the indicated times with 100nM CXCL12 at 37°C. After treatment, cells were washed twice with ice-cold PBS/0.1% BSA and CXCR4 surface expression was analyzed via flow cytometry using anti-CXCR4 PE antibody, Clone 2B11 (eBioscience, San Diego, CA).

### 2.7 Tango assay of β-arrestin mobilization

Recruitment of β-arrestin to agonist-stimulated receptors was measured using a Tango assay as previously reported [26, 27]. HTLA cells, a derivative of HEK293 cells, were transfected with either a CXCR4 D2V2-TCS-tTA or CXCR3 D2V2-TCS-tTA receptor construct that both lacked the V2 tail [26, 27]. For GRK3 over-expression, a GRK3 pcMyc_LIC plasmid was used. In addition, either yellow-fluorescent protein (YFP) or pcMyc_LIC empty-vector was used as a negative control and YFP was used as a transfection control. Briefly, HTLA cells were transiently transfected with 3 μg of receptor and 3 μg of either empty-vector control or GRK3 via calcium-phosphate precipitation in a 10 cm plate. Transfection efficiency was determined by YFP epifluorescence detection to be consistently >70%. Cells were plated in a 384-well plate (Greiner, 25,000 cells/well, 40 μL/well) in DMEM + 1% dialyzed FBS for an additional 24 hours. Cells were then serum starved for 2–4 hours in DMEM (no supplements) and followed by stimulation with human CXCL12 or human CXCL11 at indicated concentrations (highest concentration of 1 μM). After 18–24 hours, the medium was replaced with 1x BriteGlo reagent (Promega, Madison, WI, USA). Luminescence was measured on the Promega Glomax Multi + Detection System (0.5 sec/ well). Background data for each independent run was subtracted and data was normalized by setting 0% and 100% as the readout for the lowest and highest concentration of the control condition, respectively. A one-tailed Student’s t-test was used for statistical analysis at each concentration tested.

### 2.8. Chemotaxis and Chemoinvasion

Chemotaxis was performed using BD Fluoroblok 96-well chambers according to the manufacturer’s suggestions with the indicated concentrations of CXCL12 (R&D Systems, Minneapolis, MN) in the lower chamber. Non-targeted controls and GRK3-deficient cells were added to the upper chamber in 50 μl at a concentration of 2 x 10^6^/ml. Kinetic migration curve data was collected every 2 or 4 minutes and was tested for significance by a linear regression analysis (See 2.13 Statistics). Chemoinvasion of human breast cancer cells was performed using BD Matrigel 96-well chemoinvasion chambers according to manufacturer instructions. Due to reports of the low invasive capacity of 66cl4 mammary tumor cells [28], chemoinvasion for this TNBC cell line was performed at a lower matrix concentration using the Cultrex 96-well BME Cell Invasion Assay in accordance with manufacturer instructions (Trevigen, Gaithersburg, MD).

### 2.9. CXCL12 ELISA

66cl4-luc mammary tumor cells were plated at the indicated cell number per well. Supernatants were collected at 48 hours and analyzed by sandwich ELISA with a Mouse CXCL12/SDF-1 DuoSet (R&D Systems, Minneapolis, MN).

### 2.10. Proliferation assay

Cell proliferation was determined using CCK-8, Cell Counting Kit 8 (Dojindo, Rockville, MD), according to manufacturer protocol. Briefly, CCK-8 reagent was added at indicated times to cells plated at 5000 cells per well in a 96-well plate and analyzed using a standard plate reader.

### 2.11. Apoptosis assay

Assessment of anoikis was performed as previously described [29]. Briefly, 1–2 x 10^6^ 66cl4-luc GRK3-deficient and 66cl4-luc control cells were cultured either on tissue-culture treated 6-well plates or those pre-coated with poly 2-hydroxyethyl methacrylate (poly-HEMA) (Sigma, St. Louis, MO) for 20 hours at 37°C in a 5% CO_2_ incubator. Cells were harvested, washed, and assayed for apoptosis using FITC-annexin V and propidium iodide (PI) staining (Trevigen, Gaithersburg, MD). Samples were analyzed by flow cytometry.

### 2.12. *In vivo* tumor progression

Syngeneic, luciferase-tagged mouse breast cancer line 66cl4-luc was transformed with GRK3-specific knockdown shRNA or control virus. Efficacy and specificity of GRK3 knockdown was confirmed as detailed above. This cell line, as well as a control transduced with a non-target shRNA, was surgically implanted into the mammary fat pad in 6 week-old female BALB/c mice. Mice were serially monitored for tumor progression via visualization of the luciferase-tagged tumor cells on an IVIS imaging platform (Caliper Life Sciences, MA), and were sacrificed at 6 weeks post-implantation. Xenogen software was used to quantify primary and metastatic tumor burden as average radiance (p/s/cm^2^/sr).

### 2.13. Statistics

Kinetic migration curve data was compared by analysis of covariance (ANCOVA) linear regression model. See Supplemental Information for full description. Statistical significance for all other assays was determined by two-tailed t-test using GraphPad Prism version 6.00 for Windows unless otherwise noted.

## 3. Results

### 3.1. Decreased GRK3 expression correlates with basal-type breast cancer and liver metastasis in humans

**Fig 1:**
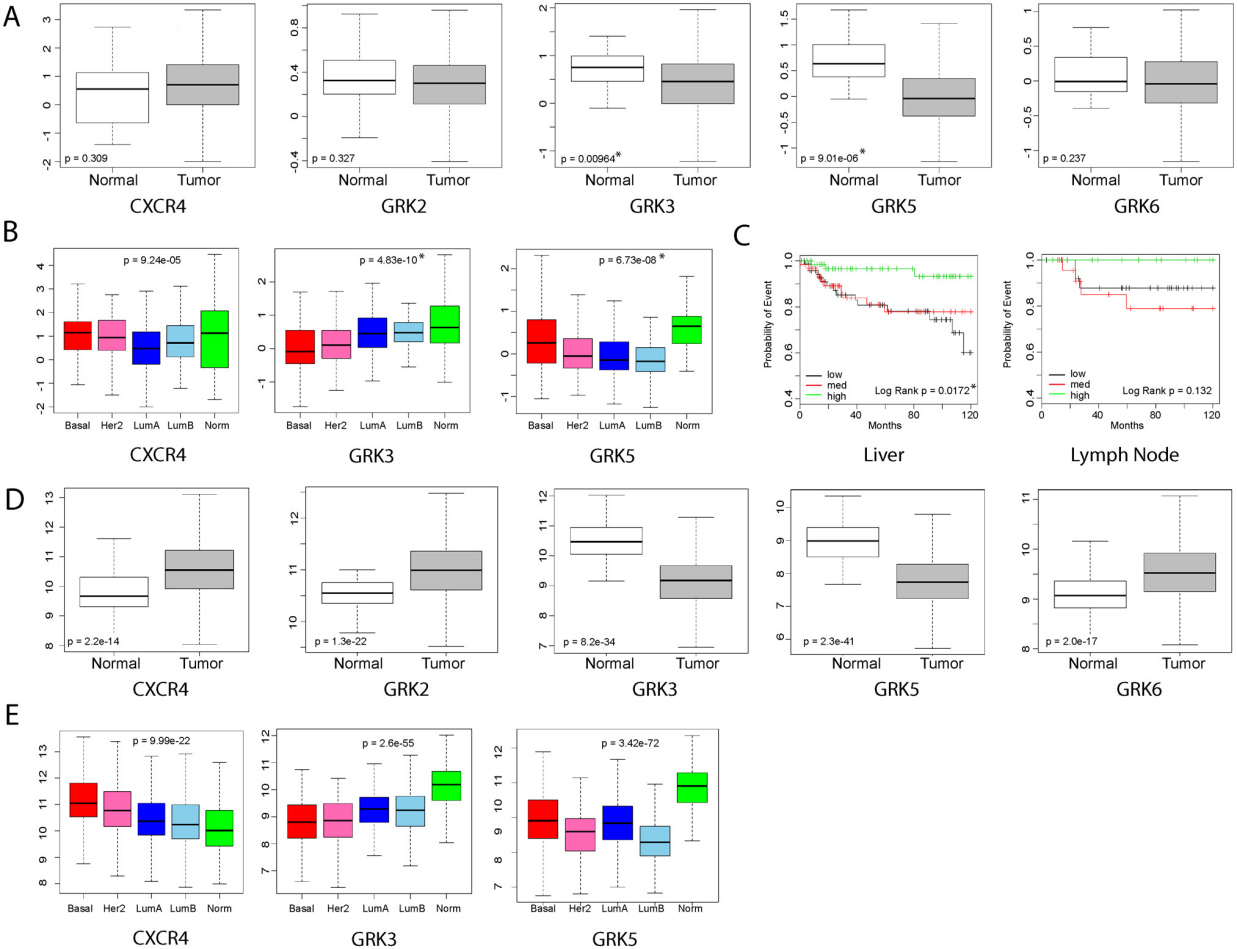
Genetic analysis of human breast cancer gene expression data associate GRK3 with tumor, basal subtype, and metastasis. Microarray datasets previously analyzed in Harrell, et al., were reanalyzed to (A) compare *CXCR4* and *GRK* expression levels between normal breast tissue and tumor tissue, (B) determine the significance of *CXCR4*, *GRK3*, and *GRK5* expression levels in human breast cancer subtypes, and (C) examine the association of *GRK3* expression in metastasis to liver and lymph nodes. Microarray data in (C) was grouped based on relative level of *GRK3* expression (low, medium, and high). (D) TCGA database was used to compare *CXCR4* and *GRK* expression levels between normal breast tissue and tumor tissue, as in (A). (E) TCGA database was used to determine changes in *CXCR4*, *GRK3*, and *GRK5* expression levels in human breast cancer subtypes as in (B). Statistical significance determined by Students t-test (A,D), ANOVA (B,E), and Log-Rank test (C).

To determine whether *GRK* expression was different between the molecular breast cancer subtypes, genomic data from the TCGA database and publicly-available databases were analyzed as previously described by Harrell, et al [6]. Analysis of public human breast cancer microarray data shows that only *GRK3* and *GRK5* isotypes are expressed at statistically significant lower levels in tumor cells compared to normal breast tissue (Fig 1A). Also from this analysis, Fig 1B shows that *GRK3* expression is lowest in the basal subtype (red box), while *GRK5* was decreased most in luminal subtypes (blue boxes). Although increased CXCR4 expression can indicate increased metastatic potential in some instances [30, 31], differential expression of *CXCR4* was not significant across these combined datasets (Fig 1A). When claudin-low and basal-type samples were grouped by *GRK3* expression level (Fig 1C), analysis of the human breast cancer data showed significant correlation of tumors expressing medium (black line) and low levels (red line) of *GRK3* to liver metastasis and a similar trend toward lymph node metastasis, while tumors expressing high levels of *GRK3* (green line) remained nearly metastasis-free. Similar results were seen in the analysis of the TCGA database (Fig 1D) where GRK3 and GRK5 again showed lower expression levels in tumor cells compared to normal breast tissue. Using this data, CXCR4 expression was shown to significantly increase in tumor cells compared to normal tissue as previously believed [30, 31]. Fig 1E shows TCGA analysis comparing breast cancer subtypes and confirms that CXCR4 expression is highest and GRK3 is lowest in basal types, though these trends are seen in all the tumor subtypes to some degree. GRK5 expression is again shown to be downregulated the most in luminal B subtype, but could also be important in other types, including basal tumors. Taken together, there is potential clinical significance for decreased *GRK3* expression in human basal breast cancer that could indicate metastatic potential.

### 3.2. GRK3 regulates CXCR4 function and the metastatic phenotype in human TNBC cell lines

**Fig 2:**
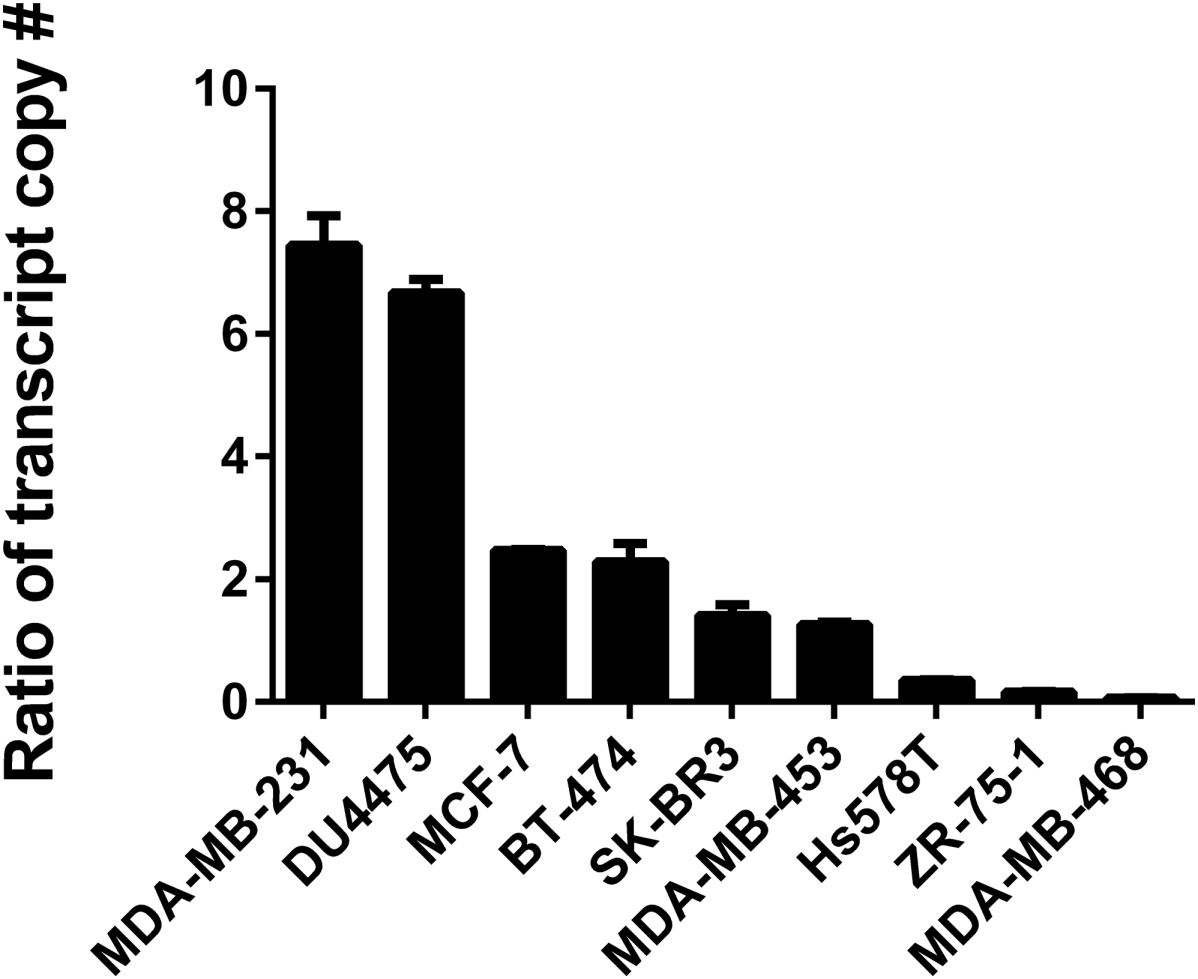
*CXCR4*:*GRK3* ratio correlates with the invasiveness of human breast cancer lines. Human breast cancer lines were analyzed by quantitative real time PCR to determine the mRNA expression levels of *GRK3* and *CXCR4*. Transcript copy number was determined using the standard curve method. Data shown are the average of two to four independent experiments. Error bars represent the SEM.

Studies by Holland and colleagues have shown that CXCR4 signaling activation, as opposed to the quantitative surface expression of CXCR4, most strongly affects metastatic behavior of human breast cancer cells [10], and this is additionally suggested by database analysis from Fig 1A. Because GRK3 negatively regulates CXCR4 function [14, 16, 17], we hypothesized that the presence of low GRK3 would be less effective at desensitizing CXCR4 signaling and would create a more aggressive metastatic phenotype. To explore this, qRT-PCR was used to establish association relationships between *CXCR4* and *GRK3* mRNA transcript levels (Fig 2) with known invasiveness of human TNBC cell lines [32, 33]. Though mRNA levels do not always correlate with protein levels, the qRT-PCR approach was undertaken as a potentially reliable prognostic assay, given the low intracellular endogenous protein expression of GRK3 and substantial cross-reactivity of antibody reagents with homologous GRK2, which is expressed in larger amounts (as shown in S1 Fig). The highly invasive breast cancer lines MDA-MB-231 and DU4475 had *CXCR4*:*GRK3* mRNA expression ratios of 7:1 and 6:1, respectively. Moderately invasive lines had ratios of 2:1 to 1:1 and the weakly invasive breast cancer lines had a *CXCR4*:*GRK3* expression ratio less than 1 (Fig 2). Because the GPCR CXCR7 also binds to CXCL12 and has been shown to have a potential a role in tumor growth and metastasis [34], we also compared *CXCR7*:*GRK3* expression ratios, but they did not correlate with invasive phenotype (S4 Fig).

**Fig 3:**
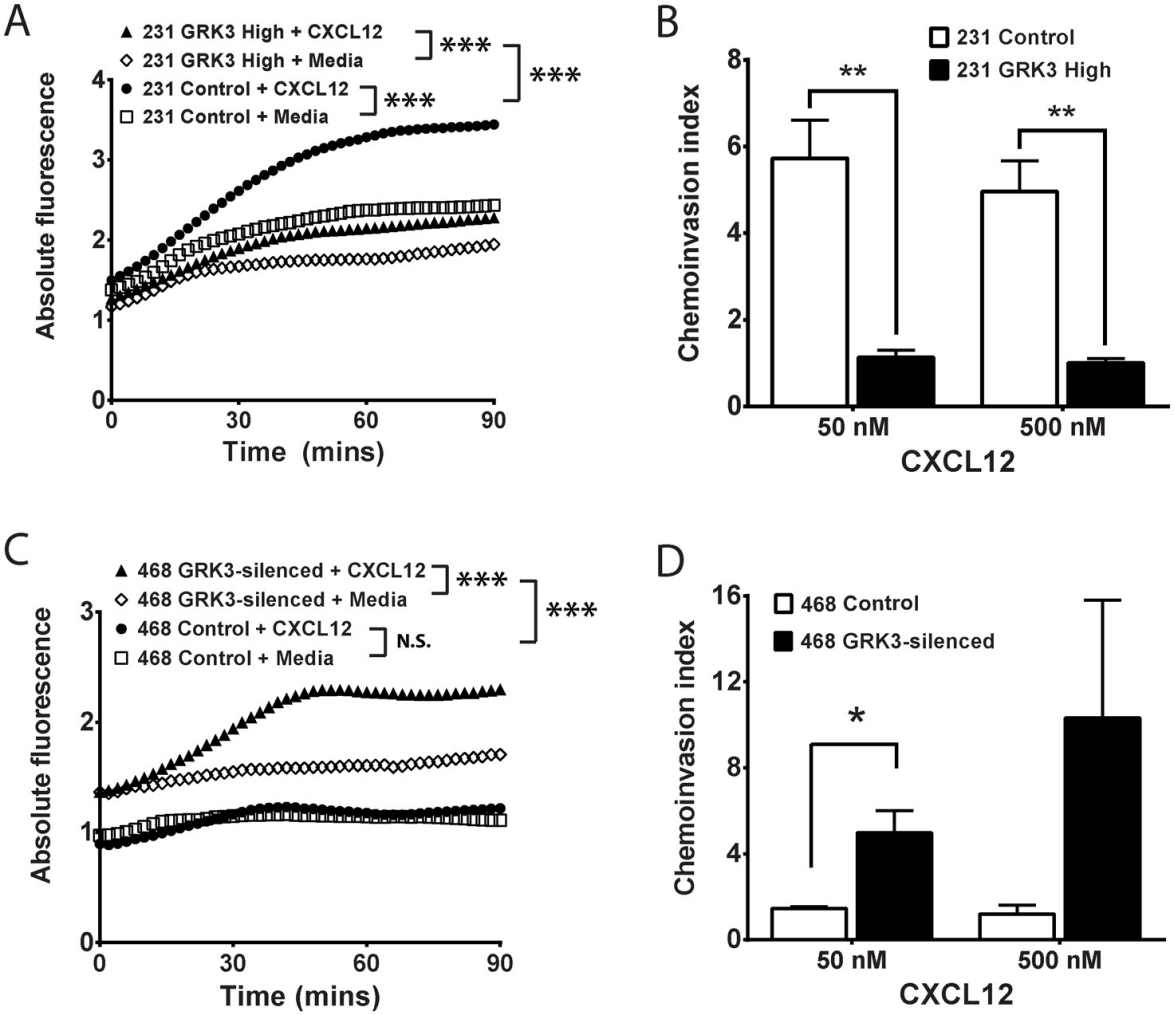
Alterations in GRK3 affect migratory responses of human breast cancer lines to CXCL12. *GRK3* expression was altered by overexpression or shRNA silencing in human breast cancer lines. (A) Chemotaxis toward media or 50 nM CXCL12 of MDA-MB-231 transiently transfected with GRK3 or control plasmid was assessed by a real-time modified Transwell assay. Transfection efficiency was routinely 40%. Results shown are the mean of 5 independent experiments. (B) MDA-MB-231 invasion through Matrigel was analyzed after 24 hours and staining with Calcien-AM. The chemoinvasion index is defined as the ratio of relative fluorescence of cells migrated toward CXCL12 over media control. Results shown are the mean of 3 independent experiments. (C) Chemotaxis toward media or 500nM CXCL12 of stably transduced MDA-MB-468 cells was assessed by a real-time modified Transwell assay. Results shown are the mean of 4 independent experiments. (D) MDA-MB-468 invasion through Matrigel was analyzed using a 96-well invasion assay. Results shown are the mean of 3 independent experiments. Error bars for all data represent the SEM. Statistical significance determined by analysis of covariance (ANCOVA) linear regression model (A and C) or by a two-tailed t-test (B and D): * p < 0.05; ** p < 0.01; *** p < 0.001; N.S. not significant.

To further define the regulatory role of GRK3 in CXCL12/CXCR4-mediated function, the *CXCR4*:*GRK3* ratios were experimentally altered by either *GRK3* overexpression or lentiviral transduced *GRK3* shRNA knockdown in the human cell lines MDA-MB-231 and MDA-MB-468, respectively. These two TNBC cell lines were chosen as comparators for being most different in intrinsic *CXCR4*:*GRK3* ratio (Fig 2) and invasive potential [32, 33, 35]. GRK3 was transiently overexpressed in the highly invasive MDA-MB-231 breast cancer cell line and confirmed by Western Blot (S2 Fig). Though both control and GRK3-overexpressing MDA-MB-231 cells migrated significantly toward CXCL12, chemotaxis toward CXCL12 was significantly inhibited by GRK3 over expression compared to control transfected cells (Fig 3A). Given that metastasis is a multistep process that involves not only migration, but also invasion, we compared control and GRK3-overexpressing MDA-MB-231 invasion through matrix. GRK3-overexpressing MDA-MB-231 cells exhibited significantly less invasion compared to controls (Fig 3B).

**Fig 4:**
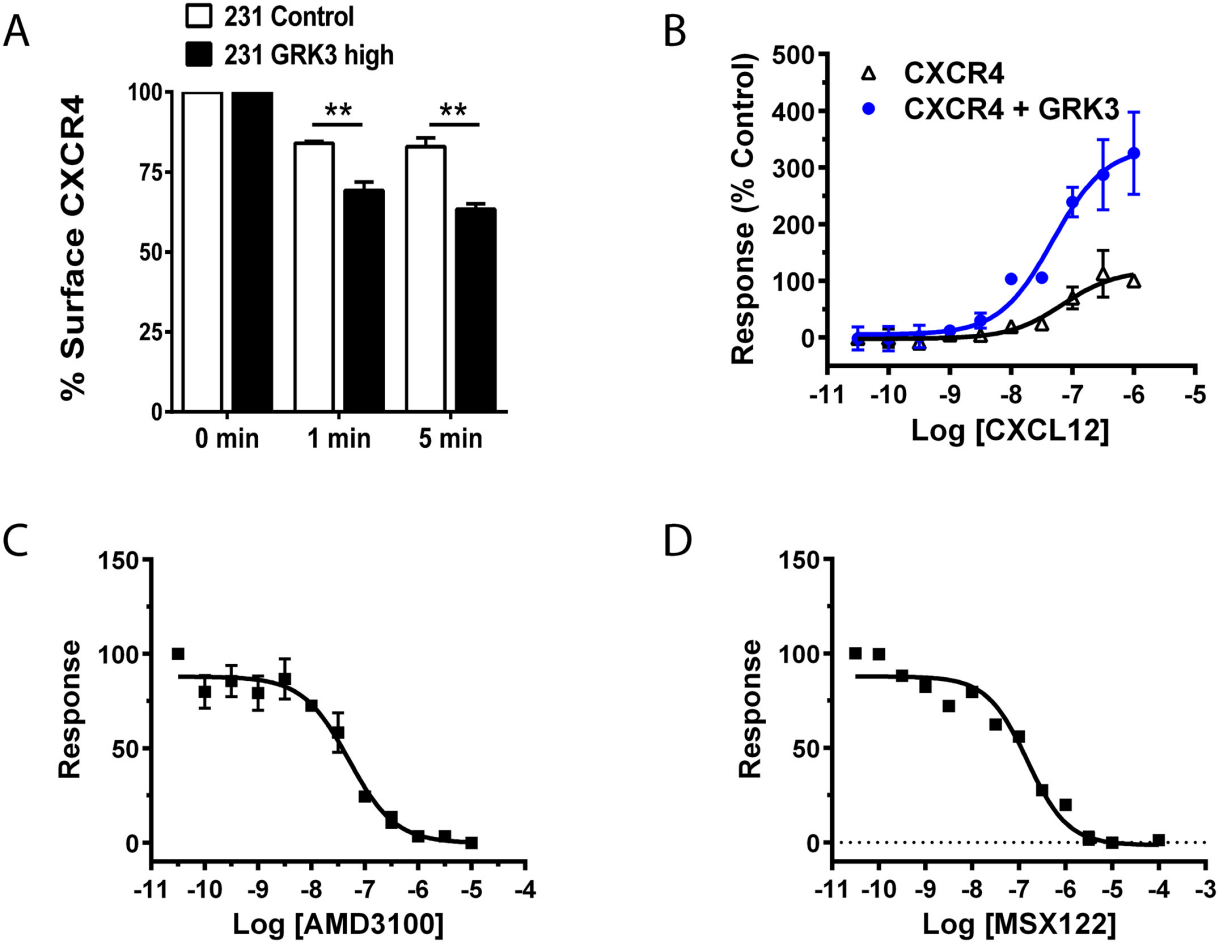
GRK3 regulates CXCL12-specific CXCR4 internalization and β-arrestin recruitment. (A) Control cells (empty plasmid) and GRK3-overexpressed MDA-MB-231 cells were treated for the indicated times with 100 nM CXCL12 at 37°C. Surface expression of CXCR4 on the surface of cells was determined by flow cytometry. Data shown are the mean of three experiments normalized to the zero time point. Error bars represent SEM. Statistical analysis was performed using a two-tailed t-test. **p < 0.01. (B) Using a TANGO arrestin-recruitment assay, HTLA cells were transfected with either CXCR4 alone or CXCR4 plus GRK3 as detailed in the Materials and Methods. Cells were plated in a 384 well plate and stimulated with CXCL12 at the indicated Molar concentrations. Luminescence was measured 24 hours post-stimulation. Error bars represent +/- SEM (n = 3). (C) TANGO results testing the CXCR4 antagonist AMD-3100. As in (B), HTLA cells were transfected with CXCR4 and GRK3 plasmids and stimulated with 10^−7^ M CXCL12 (one concentration point above EC_50_) following pre-treatment with AMD3100 at the indicated Molar concentrations. (D) TANGO results testing the CXCR4 antagonist MSX-122. HTLA cells were transfected with CXCR4 and GRK3 plasmids and stimulated with 10^−7^ M CXCL12 following pre-treatment with MSX-122 at the indicated Molar concentrations.

GRKs negatively regulates GPCR signaling by phosphorylation of terminal serine/threonine residues leading to receptor desensitization and internalization [11, 16, 17]. To confirm that overexpression of GRK3 was altering CXCL12/CXCR4 responses that culminated in increased internalization and removal of CXCR4 from the cell surface, MDA-MB-231 cells with overexpressed GRK3 were compared to controls and had significantly more internalization of CXCR4 receptor in response to CXCL12 over time (Fig 4A).

GPCR internalization following GRK phosphorylation is mediated by beta-arrestins [12, 36, 37].

To show how GRK3 can influence CXCR4 internalization by recruiting β-arrestin, we used the TANGO assay as described in the Materials and Methods. Fig 4B shows the CXCL12 concentration-dependent internalization of CXCR4 (black open triangles), which is significantly enhanced by the addition of GRK3 (blue closed circles). Known CXCR4 inhibitors AMD3100 (Fig 4C) and MSX-122 (Fig 4D) inhibit CXCL12-mediated arrestin recruitment in a concentration-dependent manner. By comparison, the cancer-relevant receptor CXCR3, which has been shown to have functional interactions with CXCR4 [38–40], recruits β-arrestin in the TANGO assay when stimulated with its ligand CXCL11, but the response is not enhanced by GRK3 (S5A Fig). CXCL12 stimulation of CXCR3 does not recruit β-arrestin, as expected (S5B Fig).

In contrast to MDA-MB-231, MDA-MB-468 are weakly-metastatic TNBC cells with a low *CXCR4*:*GRK3* expression ratio (Fig 2), suggesting that GRK3 may be mitigating migration and invasion through increased desensitization/internalization of CXCL12-activated CXCR4. To test this hypothesis, GRK3 was stably knocked down by lentiviral shRNA expression in MDA-MB-468 cells and confirmed by Western Blot (S2 Fig). Control MDA-MB-468 cells did not show statistically significant migration toward CXCL12. GRK3 knockdown in MDA-MB-468 cells significantly increased *in vitro* chemotaxis (Fig 3C) and chemoinvasion (Fig 3D) toward CXCL12, thereby making their migratory phenotype more analogous to the aggressive MDA-MB-231 line. Taken together, the results indicate an important role for GRK3 in the regulation of CXCL12/CXCR4-mediated functions and in the migratory phenotypes of human TNBC cell lines.

### 3.3. GRK3 gene silencing potentiates mammary tumor establishment and metastasis *in vivo*

**Fig 5:**
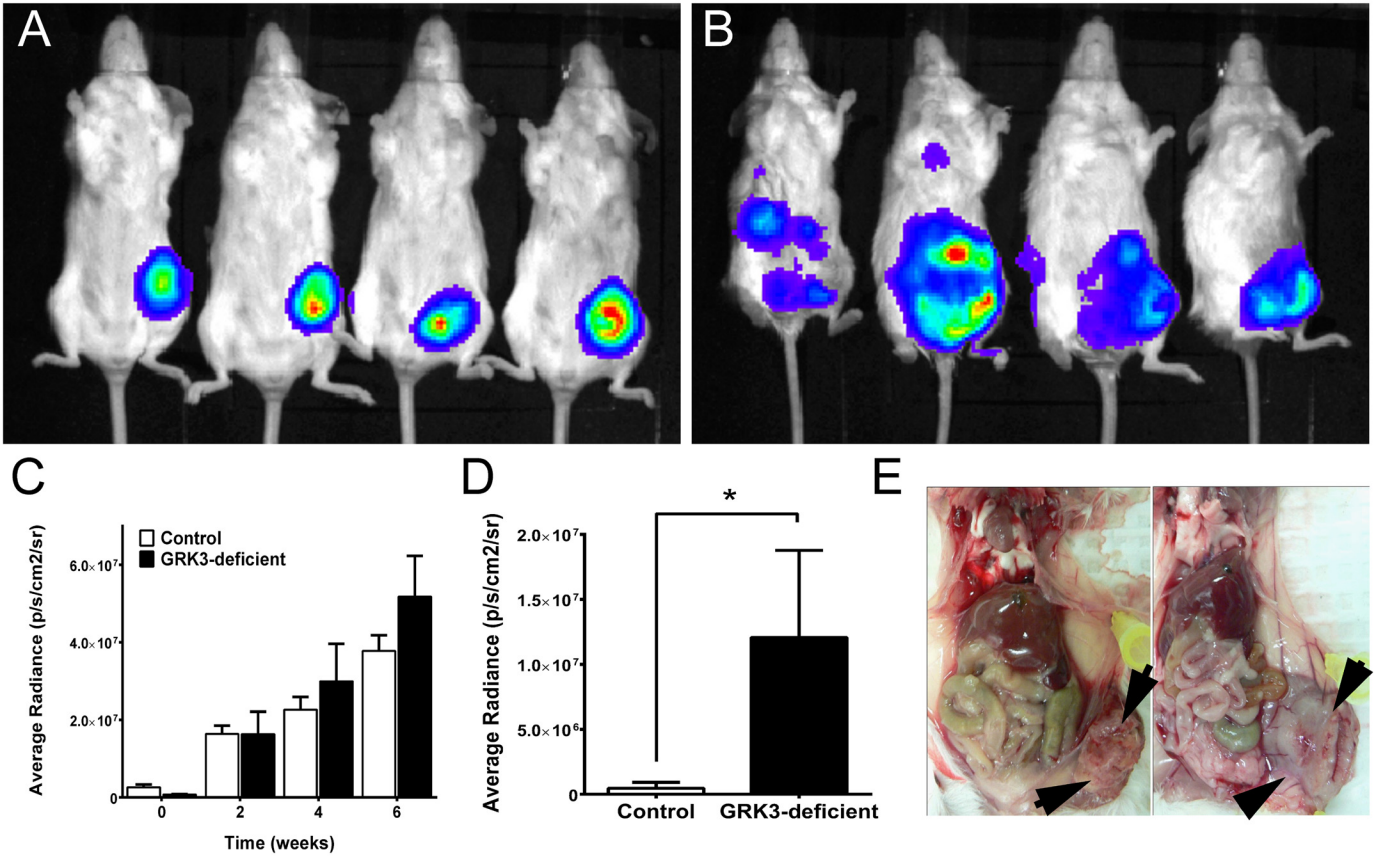
Increasing the *CXCR4*:*GRK3* ratio in 66cl4-luc mammary tumor cells increases *in vivo* metastasis. (A) Balb-c mice with 66cl4-luc control transduced cells visualized by optical imaging at 6 weeks post-implantation demonstrate only primary tumor growth in contrast to mice with 66cl4-luc GRK3 deficient cells (B), which show extensive and distant metastasis. Images shown in *C*. and *D*. are representative of two independent experiments. Quantification of total (C) and metastatic (D) tumor in control versus GRK3-deficient tumors as imaged by luciferase activity. Data shown in (C) are n = 10 mice at weeks 0 and 2, n = 9 week 4, n = 7 week 6 (due to disease mortality). Data in (D) are n = 8 for Control, n = 7 for GRK3-deficient. (E) Necropsy of Balb-c mouse with control 66cl4-luc tumor cell implant showing small, encapsulated primary tumor (left panel). Necropsy of animal with GRK3-silenced 66cl4-luc tumor cell implant demonstrating larger, friable primary tumor and prominent neovascularization (right panel). All error bars represent SEM.

To assess the role of GRK3 in metastasis *in vivo*, a syngeneic, immunocompetent mouse model of TNBC [21] was used. The mouse mammary tumor line 66cl4-luc is a weakly-metastatic, BALB/c tumor line expressing a luciferase construct that allows for tracking by *in vivo* optical imaging [24]. *GRK3* mRNA and protein levels were silenced in the 66cl4-luc line by lentiviral shRNA transduction (S1 Fig) in an analogous fashion to silencing GRK3 in the non-invasive human MDA-MB-468 cells. Non-targeted control and GRK3-deficient 66cl4-luc cells were surgically implanted into the mammary fat pads of BALB/c mice and monitored by optical imaging for tumor growth and metastasis for six weeks, and organ-specific metastatic tumor burden was examined in detail by histopathology at experiment termination. Non-target control 66cl4-luc cells established primary tumors *in vivo*, but extended few metastases (Fig 5A and Table 1). In contrast, mice implanted with 66cl4-luc GRK3-deficient cells developed large primary tumors and distant metastases (Fig 5B and Table 1). Fluorescent intensity was analyzed with Xenogen software and reported as average radiance (p/s/cm^2^/sr) to quantify primary and metastatic tumor burden over time. The analysis determined that primary tumor burden was not significantly different, albeit slightly increased on average, in GRK3-deficient tumors compared to controls throughout the 6-week period (Fig 5C). More notably, mice implanted with 66cl4-luc GRK3-deficient cells developed significantly more metastases at experiment termination (Table 1 and Fig 5D). Histological data confirmed GRK3-deficient 66cl4 cells metastasized mostly to the lung, consistent with the model [28], and additionally to distant organ sites, such as liver, at a higher frequency (Table 1). Along with the optical imaging data, necropsy of primary tumors showed that GRK3-deficient tumors displayed a different anatomy (Fig 5E), suggesting that these tumors may appear larger due to cell movement and resemble an invasive phenotype [41]. These *in vivo* data suggest that the metastasis of TNBC cells can be altered when the negative signaling regulator GRK3 is affected and that the sites of metastasis correlate with human data (Fig 1) as well as where CXCL12 is expressed.[7–9]

**Table 1:** GRK3-deficient 66cl4-luc mammary tumors disseminate distant metastasis.

Primary Tumor	Liver Metastasis	Lung Metastasis	Spleen Metastasis	Adrenal/Kidney Metastasis	Pancreas Metastasis	Skeletal Muscle Metastasis
GRK3 kd		**X**			**X**	
GRK3 kd		**X**				**X**
GRK3 kd	**X**	**X**			**X**	**X**
GRK3 kd		**X**				
GRK3 kd	**X**	**X**	**X**	**X**		**X**
GRK3 kd	**X**	**X**		**X**		
Control						
Control	**X**					**X**
Control						
Control		**X**				
Control						
Control	**X**	**X**	**X**			
Control						

### 3.4. Silencing GRK3 expression does not alter cell proliferation, CXCL12 secretion, or anoikis

**Fig 6:**
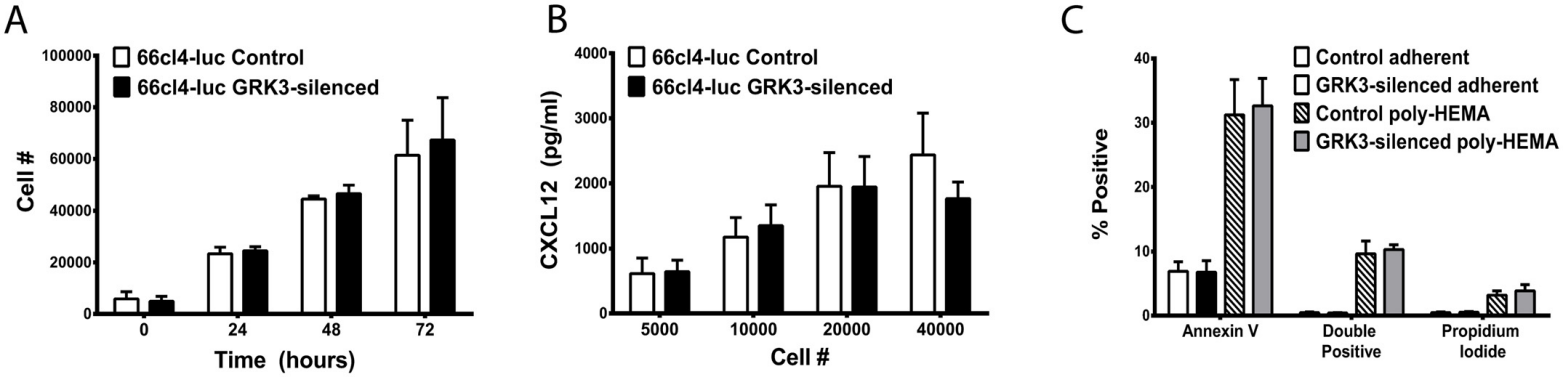
GRK3 deficient and control 66cl4-luc cells secrete soluble CXCL12, proliferate, and undergo apoptosis similarly. (A) Viable cell density was measured by colormetric assay (Cell Counting Kit-8, Dojindo) at indicated times and cell number estimated by standard curve (n = 3 + SEM). (B) Cells were plated in triplicate at the indicated number per well and incubated in culture conditions for 48 hours. CXCL12 levels in the supernatants were determined by sandwich ELISA and quantified by standard curve (n = 3; mean + SEM). (C) GRK3-deficient and control 66cl4-luc cells were incubated in adherent versus detached (poly-HEMA) conditions overnight and analyzed for apoptosis by flow cytometry for Annexin V and propidium iodide staining. Data is the mean of 4 independent experiments (error bars = SEM).

GRK3-deficient 66cl4-luc tumors appear larger in diameter than control tumors when visualized by optical imaging and necropsy (Fig 5), and studies by Woerner and colleagues have described an important role for GRK3 in regulating primary tumor growth in glioblastoma [14]. Since growth rates could impact metastatic potential in breast cancer, an *in vitro* assay was performed to assess intrinsic proliferation rates of GRK3-deficient and control 66cl4-luc cells. No significant difference was found in the proliferation of these 66cl4-luc cells in *in vitro* cultures at baseline (Fig 6A) or in the presence of ligand CXCL12 or CXCR4-antagonist, AMD3100 (S6 Fig).

Autocrine stimulation from secreted CXCL12 has been shown to influence tumor behavior and phenotype [42], and CXCL12 expression in breast cancer cells has been correlated with decreased metastatic potential in humans [43]. To examine CXCL12 secretion from control and GRK3 deficient 66cl4-luc cells, CXCL12 in supernatants was measured by ELISA but was not found to be different between cell types (Fig 6B).

Epithelial cells, including normal breast epithelium, are dependent upon contact with underlying extracellular matrix for survival and undergo apoptosis in response to detachment, a process termed anoikis. Resistance to anoikis is a hallmark of metastatic progression in cancer [44]. Given that CXCR4 signaling is known to regulate anoikis in tumor progression [44, 45], 66cl4-luc GRK3-deficient cells were compared to 66cl4-luc control cells for their apoptotic response to detachment; data showed that there was no intrinsic difference in anoikis between the different 66cl4-luc tumor types *in vitro* (Fig 6C).

### 3.5. GRK3 deficiency increases migration of 66cl4-luc mammary tumor cells to CXCL12

**Fig 7:**
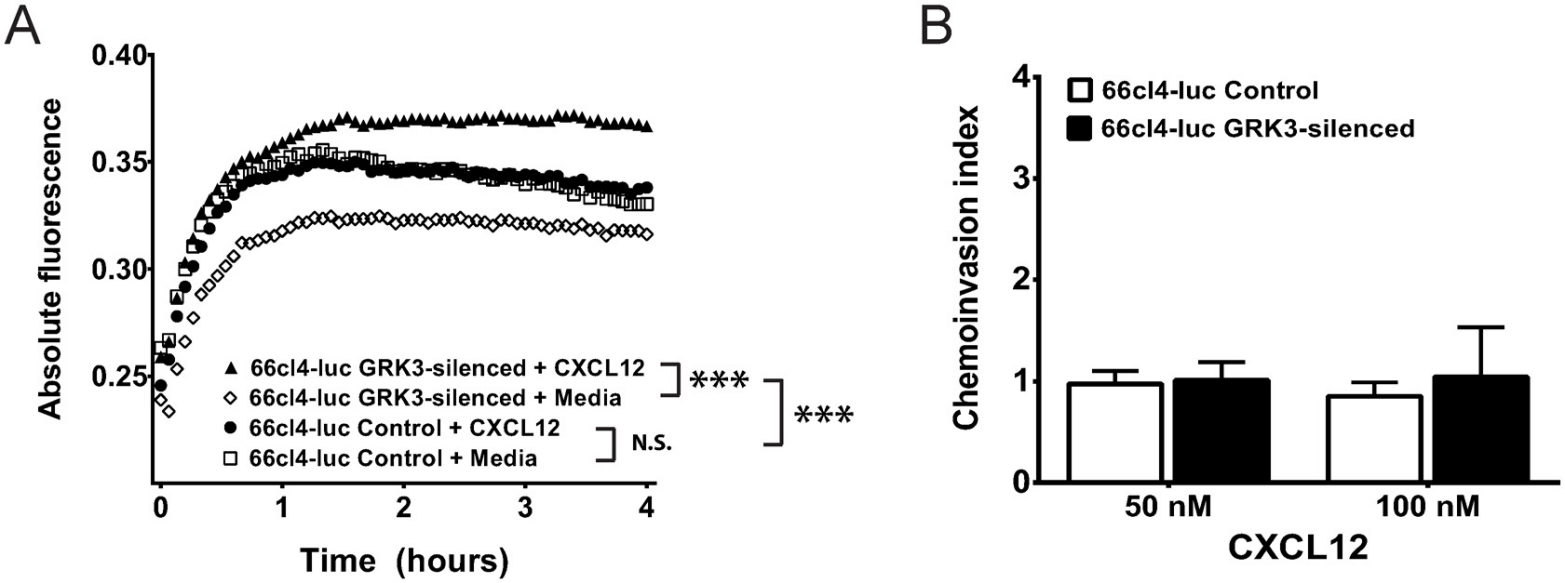
GRK3-deficient 66cl4-luc mammary tumor cells have increased chemotaxis but not invasion. GRK3 was silenced by lentiviral shRNA in 66cl4-luc cells to examine the effect on migration. 100,000 cells were added per well to the top of a 96-well Fluoroblok Transwell plate, and 12.5nM (100ng/ml) CXCL12 or media was in the lower chamber. Migration was monitored over 4 hours using a Fluoroskan Ascent FL plate reader. (A) Control transduced 66cl4-luc cells do not migrate toward CXCL12 (closed circle) over media control (open square) whereas GRK3 deficient 66cl4-luc cells display directional chemotaxis toward CXCL12 (closed triangle) over media control (open diamond). Statistics significance determined by analysis of covariance (ANCOVA) linear regression model: *** p < 0.001; N.S. not significant. (B) Control and GRK3 deficient 66cl4-luc cells were loaded into the upper chamber of Cultrex invasion plates in triplicate (see Materials and Methods). After addition of media alone or CXCL12 (50 or 100 nM) to the lower chamber, cells were incubated for 24 hours, stained with Calcein, and then counted for invasion into matrix (n = 3 ± SEM).

The analysis of human breast cancer cell lines in Figs 2 and 3 suggests that cells with higher *CXCR4*:*GRK3* ratios are more metastatic because of increased migration and invasion toward CXCL12. Additionally, the *in vivo* 66cl4-luc model shows increased distant metastasis of tumors to sites known to express high CXCL12 [7–9] after GRK3 silencing by lentiviral shRNA (Fig 5 and Table 1). To determine whether GRK3-deficient 66cl4-luc metastasis could result from enhanced CXCL12/CXCR4-mediated migration, a kinetic chemotaxis assay was performed as previously described. Control 66cl4-luc cells did not migrate to CXCL12 over media alone, while GRK3-deficient 66cl4-luc cells showed significantly enhanced directional migration to CXCL12 (Fig 7A). Because 66cl4 cells are thought to perform poorly in standard *in vitro* invasion assays similar to those used for human cells in Fig 3 [28], a Cultrex 96-well tumor invasion plate was coated with a lower concentration basement membrane extract (0.2X BME) to assess *in vitro* invasion. While detectable amounts of cells invaded the Matrigel, addition of CXCL12 did not significantly increase the invasiveness of either control cells or GRK3 deficient 66cl4-luc cells over media alone (Fig 7B). Because breast cancer cells can have different behaviors and phenotypes in 3-dimensions versus 2-dimensions, 66cl4-luc cells were also tested in a 3D system of invasion (S7 Fig) with negative results similar to the 2D Transwell invasion assay.

## 4. Discussion

**Fig 8:**
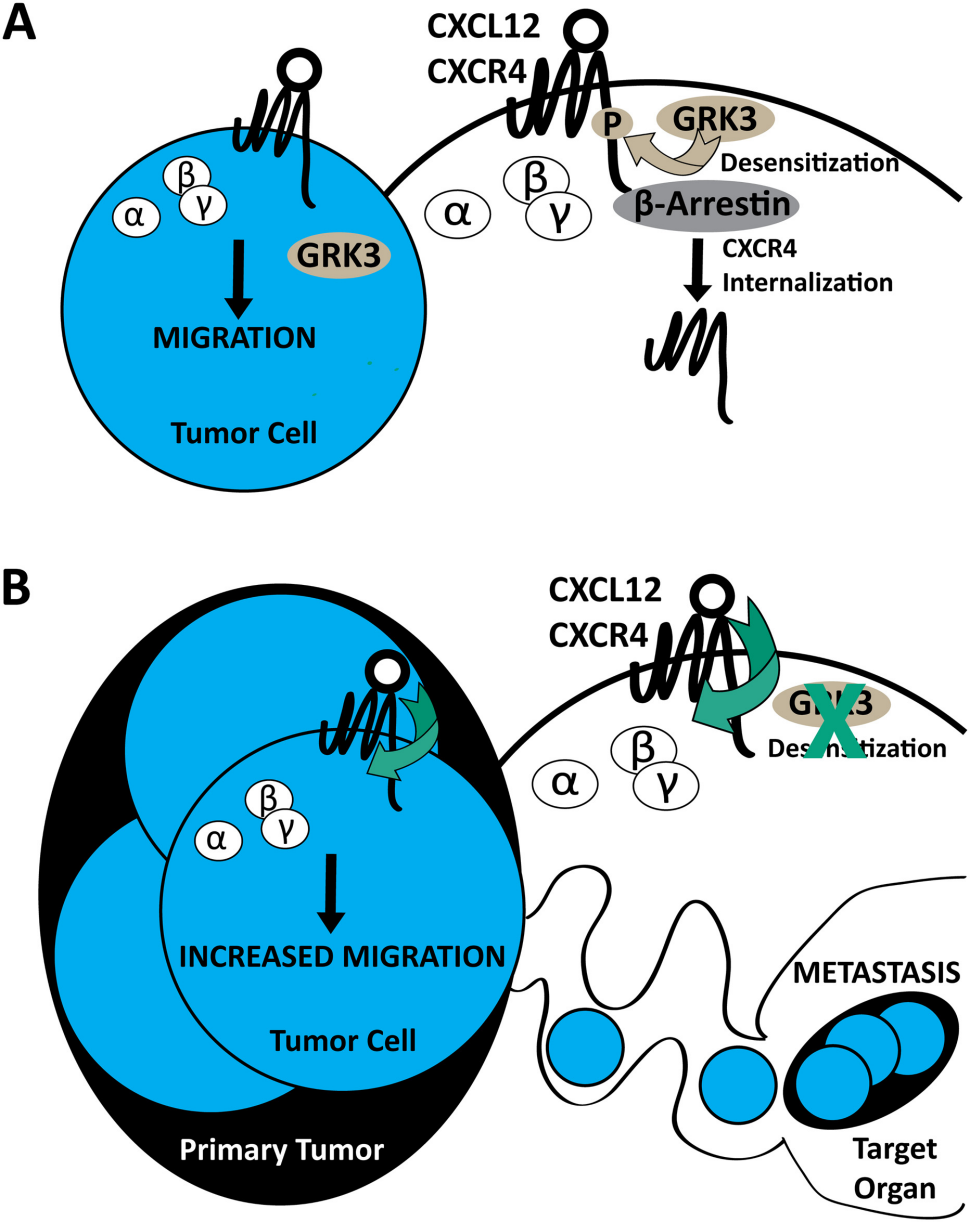
Summary model of GRK3 regulation of CXCR4-driven metastasis. Extracellular ligand stimulation of CXCL12 on G protein-coupled receptor (GPCR) CXCR4 elicits conformational changes of receptor, activation and dissociation of guanine nucleotide binding proteins, and downstream signaling for tumor cell migration (A). Negative regulation of surface receptor expression is mediated by G protein-coupled receptor kinase 3 (GRK3), which phosphorylates the carboxyl terminus of CXCR4 for desensitization, thus prompting β-arrestin recruitment for receptor internalization. The presence of GRK3 limits ligand/receptor signaling by contributing to desensitization and by reducing CXCR4 surface expression (A, inset). Upon GRK3 deficiency, CXCR4 receptor expression is enhanced thus allowing increased opportunities for CXCL12 extracellular ligand/receptor stimulation, signaling, and migration (B). The absence of GRK3 enhances ligand/receptor signaling by prolonging CXCR4 surface expression (B, inset).

Breast cancer metastasis is known to be mediated in part by the CXCL12/CXCR4 signaling axis [7]. We now add G protein coupled receptor kinase 3 (GRK3) as an important regulator, specifically with respect to invasion and metastasis, in human breast cancer tissue, TNBC cell lines, and in an immunocompetent mouse model. Previous work by others has suggested that quantitative levels of CXCR4 surface expression, as well as CXCL12 concentration and availability, can affect the metastatic phenotype [9]. Recent evidence points to an even greater importance for CXCR4 expression in TNBC, which contributes to tumor size and metastatic potential as well as predicts poor prognosis in terms of overall and disease-free survival [46–48]. However, further experiments have clarified that the magnitude of CXCR4 signaling, as perturbed by gene silencing, mutation, overexpression, or antagonism, is a key factor that affects increased migration, invasion, and metastasis [7–9, 30, 49]. In support of this argument, Holland et al. showed that the absolute level of CXCR4 expressed on the surface of breast cancer cells alone did not accurately reflect or predict metastatic potential [10]. The data we present offers a mechanistic explanation for the role of CXCR4 signaling in breast cancer metastasis. The data show that relative GRK3 expression correlates with tumorgenicity, invasiveness, and metastatic potential in both human (Figs 1, 2 and 3) and mouse models (Fig 5 and Table 1) and further, that manipulating GRK3 levels directly affects the chemotactic and metastatic potential of breast cancer cells (Figs 3 and 7). Thus, we propose that high CXCR4 on the cell surface, combined with decreased intracellular regulatory GRK3, leads to impaired receptor internalization and more active CXCL12-mediated migration of the cancer cell (Fig 8).

We acknowledge that GRK3 in breast cancer is unlikely to be affecting CXCL12/CXCR4 interactions exclusively *in vivo* and could certainly be affecting additional chemokine receptors such as CXCR7 [50–52], CXCR3 [53, 54], CCR7 [55–57], and others.[58] However, our data support a strong role for GRK3-mediated regulation of CXCL12 metastatic responses, arrestin-mediated recruitment, and CXCR4 receptor internalization *in vitro* (Figs 3, 4 and 7). In contrast, there is less compelling data for GRK3-mediated regulation of CXCR3 where we show that arrestin recruitment is independent (S5 Fig), and there was a lack of correlational expression data supporting *CXCR7*:*GRK3* to the metastatic phenotype (S4 Fig). As genotyping has become an important tool to provide the prognosis and treatment of TNBC [59], the relative gene expression of markers such *CXCR4* and *GRK3* may provide additional prognostic information as it pertains specifically metastasis.

The metastatic potential of breast cancer cells is acutely sensitive to regulation of the CXCL12/CXCR4 signaling axis governed by receptor expression, desensitization, internalization, and recycling dynamics [9, 18, 19]. These properties of CXCR4 are directly influenced by phosphorylation by GRKs and subsequent downstream components, such as β-arrestins [16, 17]. GRK3 has previously been shown to regulate tumor growth and proliferation in glioblastoma (GBM). GRK3 expression is down-regulated by EGFR signaling in GBM compared to normal astrocytes, which leads to a corresponding increase in tumor growth.[14] Published data by others have also shown that down-regulation of GRK6 in medulloblastoma [15] and Lewis lung carcinoma [60] can similarly regulate tumor size, migration, and metastasis. In contrast, our results correlate GRK3 with breast cancer metastatic potential via regulation of tumor cell migration and invasion (Figs 3 and 7) without significant effect on tumor growth or detachment-induced death (Fig 6). These differences could potentially be explained by different cell types having altered GRK-specific phenotypes or redundancy based on tissue expression and/or targeted GPCR-mediated regulation.

*In vivo* bioluminescent imaging of 66cl4-luciferase tumors demonstrates an apparently larger, more diffuse tumor arising from GRK3-knockdown cells (Fig 5) despite similarity of cellular radiance quantification. One potential explanation is that the density of the tumor might be affected by the mobility of the individual GRK3-deficient tumor cells or by the recruitment of non-tumor cells into the tumor site. GRK3-deficient tumors also contained notable areas of necrotic tissue (data not shown). Since necrotic cells would no longer luminesce, it is also possible that GRK3-deficient tumors did proliferate more *in vivo* in response to environmental cues not present in the *in vitro* assay, and that resultant tissue necrosis confounded optical quantification. The appearance of necrotic regions in primary tumors can also contribute to increased metastases [61, 62]; however, this hypothesis was not directly tested in this study and remains a future endeavor.

High levels of CXCL12 known to exist in metastatic target tissues [7] raise the possibility that the functional outcome of altered GRK3 expression occurs distally from the primary tumor. Since 66cl4-luc cells are weakly invasive and this phenotype is unaltered by *GRK3* gene silencing (Fig 7B), we propose that the observation of increased metastases of GRK3-deficient 66cl4-luc cells is due to increased migration (Fig 7A), possibly *within* the tumor microenvironment, leading to higher rates of intravasation that are controlled by other mechanisms. This interpretation is consistent with the larger, diffuse tumors imaged in mice implanted with 66cl4-luc GRK3-deficient cells (Fig 5). The sprawling, less dense tumor mass and increased chemotactic ability of the GRK3-deficient 66cl4-luc cells suggest that more motile cells within the primary tumor ultimately result in the increased distant metastases. However, our human cell line data do show an invasive phenotype, thus the findings using the 66cl4-luc cell line may be species and/or tumor specific. Future studies examining the *in vivo* and *in vitro* migratory, invasive, and metastatic potential of human derived breast cancer cells will be needed to see if these present results are generalizable to a broader array of malignant phenotypes.

CXCL12 binds to CXCR7 as well as CXCR4 [34]. CXCR7 is a dual-specificity GPCR that binds to CXCL12 with higher affinity than CXCR4 and also binds to the CXCR3 ligand, CXCL11, with low affinity. CXCR7 may function as a regulator of CXCR4 signaling by heterodimerization or as a decoy receptor, scavenging CXCL12 without leading to traditional G protein signals [63]. In addition, CXCR7 is thought to have G protein independent signaling functions through beta-arrestin mediated signals [39, 64]. CXCR7 in breast cancer cells can affect tumor growth, survival, and adhesion to vasculature [65]. Furthermore, CXCR7 can also influence CXCR4-mediated chemotaxis and invasion, as well as independently regulate tumor growth by promoting angiogenesis [34]. The breast cancer cells used in the current study failed to demonstrate a correlation between CXCR7 levels and tumor cell migration or metastatic potential (S4 Fig). This may be due to low levels of CXCR7 expression in these cells. Furthermore, current data implicate GRK2, not GRK3, as a potential regulator of arrestin-biased signaling through CXCR7, which fails to activate classic G protein pathways [64, 66, 67]. These authors acknowledge that GRK3 likely regulates other GPCRs that have relevance to cancer [14, 68, 69]; in agreement with their speculation, the data presented herein describe an important functional role for GRK3 in regulating CXCR4 that impacts metastatic breast cancer progression.

Most TNBC is associated with high CXCR4 expression, increased metastatic potential, and poor patient prognosis [47, 48]. Our data emphasize the functional relationship of GRK3 as it pertains to CXCL12/CXCR4 migration in breast cancer and specific molecular subtypes. The analysis of human genetic data confirms potential involvement of GRK3 in tumor progression and metastasis (Fig 1). Across large datasets, decreased *GRK3* expression correlated better with tumor (vs normal breast) than changes in *CXCR4* expression. In our resected mouse mammary tumors, the *CXCR4*:*GRK3* expression ratio was stable even after 6 weeks of tumor implantation and distant metastasis (S2 Fig), suggesting that the *CXCR4*:*GRK3* ratio is preserved and could be a useful prognostic indicator at extended times during the disease course. Others have proposed CXCR4 signaling and regulatory pathways as an attractive target for breast cancer treatment [8, 10, 48, 70]. Considering the importance of GRK3 regulation of breast cancer migration, invasion, and metastasis described in this manuscript, understanding which tumors have more dysregulated signaling through CXCL12/CXCR4 could be used as a biomarker to predict which patients might respond better to CXCR4 antagonism or as an alternative, targeted therapy directed specifically at GRKs.

## Supporting Information

S1 Fig Altering the *CXCR4*:*GRK3* in 66cl4-luc mammary tumor cells by *GRK3* shRNA silencing. (A) 66cl4-luc murine mammary tumor cells were stably transduced with lentiviral GRK3 shRNA or non-target control plasmids and were analyzed by qRT-PCR to determine the mRNA expression levels of *GRK3* and *CXCR4* after normalization to the IDUA housekeeping gene. Data is expressed as a ratio of CXCR4 to GRK3. 66cl4-luc Control n = 3, 66cl4-luc GRK3-deficient n = 4. (B) Representative GRK3 Western blot showing shRNA silenced GRK3-deficient 66cl4-luc cells compared to controls after immunoprecipitation. Blots were stripped and reprobed to confirm equal loading. Shown is actin blot of IP supernatant lanes. (C) Prior to implantation into Balb/c mice for *in vivo* studies, GRK3 is silenced approximately 50–60% in 66cl4-luc cells versus control as determined by qRT-PCR (n = 4). (D) GRK3 gene silencing was validated at 6 week experiment termination by qRT-PCT (n = 4). GRK3 mRNA expression was normalized to IDUA housekeeping gene and relative % expression determined by ΔΔC_T_ method. All error bars represent SEM.
(TIF)

S2 Fig Altered GRK3 protein expression in human breast carcinoma lines. (A) Western blot depicts GRK3 protein overexpression in MDA-MB-231 cells; lysates were also blotted for actin as a loading control. (B) GRK3 Western blot of MDA-MB-468 cell lysates that were stably transduced with lentiviral non-target control plasmid (NT) or GRK3 shRNA (GRK3 silenced). Lysates of equal protein concentration (determined by Protein BCA assay) were immunoprecipitated using anti-human GRK3 monoclonal antibody (Abgent). Immunoprecipitation samples, top, show GRK3 protein knockdown in the shRNA-silenced MDA-MB-468 cells. GADPH in IP supernatants, bottom, confirm equal loading.
(TIF)

S3 Fig Surface receptor expression is not altered following stable GRK3 shRNA knockdown. Human breast cancer line MDA-MB-468 (A, C) and murine breast cancer line 66cl4-luc (B, D) that have been stably transduced with GRK3 shRNA (red) or a control sequence (blue) and were stained for CXCR4 and CXCR3 and analyzed by flow cytometry. For human cells, antibodies used were: mouse anti-human CXCR4-PE (clone 12g5, isotype mouse 2a PE, Biolegend), anti-human CXCR3-APC (clone 49801, isotype mouse G1 APC, R&D Systems), and for mouse cells, rat anti-mouse CXCR4-PE (clone 2b11, isotype rat 2b PE, eBioscience) and rat anti-mouse CXCR3-APC (clone 220803, isotype rat 2a, R&D Systems). Negative controls were stained using equivalent amounts of isotype color controls. Shown are representative histograms of 3 independent experiments.
(TIF)

S4 Fig *CXCR7*:*GRK3* relative copy number does not correlate with the metastatic potential of human breast cancer lines. Human breast cancer lines (in descending order moving from left to right of most highly metastatic to least metastatic) were analyzed by quantitative real time PCR to determine the mRNA expression levels of *GRK3* and *CXCR7*. Transcript copy number was determined using the standard curve method. Data shown are the average of two to four independent experiments. Error bars represent the SEM.
(TIF)

S5 Fig CXCR3 recruits arrestin independently of GRK3 in response to CXCL11 and does not recruit arrestin in response to CXCL12. (A) Using a TANGO arrestin-recruitment assay, HTLA cells were transfected with either CXCR3 alone or CXCR3 plus GRK3 as detailed in the Materials and Methods. Cells were plated in a 384 well plate and stimulated with CXCL11 at the indicated Molar concentrations. Luminescence was measured 24 hours post-stimulation. Error bars represent +/- SEM (n = 3). (B) HTLA cells were transfected with either CXCR3 alone or CXCR3 plus GRK3 as in (A). Cells were plated in a 384 well plate and stimulated with CXCL12 at the indicated Molar concentrations. Luminescence was measured 24 hours post-stimulation. Error bars represtent +/- SEM (n = 3).
(TIF)

S6 Fig Addition of CXCL12 or AMD3100 does not alter the proliferation of 66cl4-luc cells over 72 hours. Viable cell density was measured by colormetric assay (Cell Counting Kit-8, Dojindo) at indicated times and cell number estimated by standard curve (n = 2 + SEM). 66cl4-luc non-target control (A) and GRK3-silenced (B) cell proliferation was tested with or without exogenous CXCL12 (100 ng/ml) added to the culture media. Since 66cl4-luc cells make large amounts of endogenous CXCL12 (Fig 6), 66cl4-luc non-target control (C) and GRK3-silenced (D) cell proliferation was also tested in presence or absence of CXCR4 antagonist AMD3100 (5 μg/ml).
(TIF)

S7 Fig 66cl4-luc cells do not invade in a 3D invasion assay regardless of CXCL12 stimulation or GRK3 deficiency. 66cl4-luc murine breast cancer cells were tested using the Cultrex 3D Spheroid Cell Invasion Assay (Trevigen) according to manufacturer’s suggestions. Briefly, cells were allowed to assemble into spheroids for 3 days. Invasion matrix and media (+/- CXCL12) were added and images captured (invasion Day 0). Images were captured at the days indicated and analyzed using Image J software as described in the product insert. Data shown is the mean of two independent experiments (error bars +/- SEM).
(TIF)

S1 Methods Supplemental Information. (DOCX)
