# Supplementary figures and images for: G Protein Coupled Receptor Kinase 3 Regulates Breast Cancer Migration, Invasion, and Metastasis
Source: PLoS One. 2016 Apr 6;11(4):e0152856. doi: 10.1371/journal.pone.0152856 (PMC4822790; doi:10.1371/journal.pone.0152856)

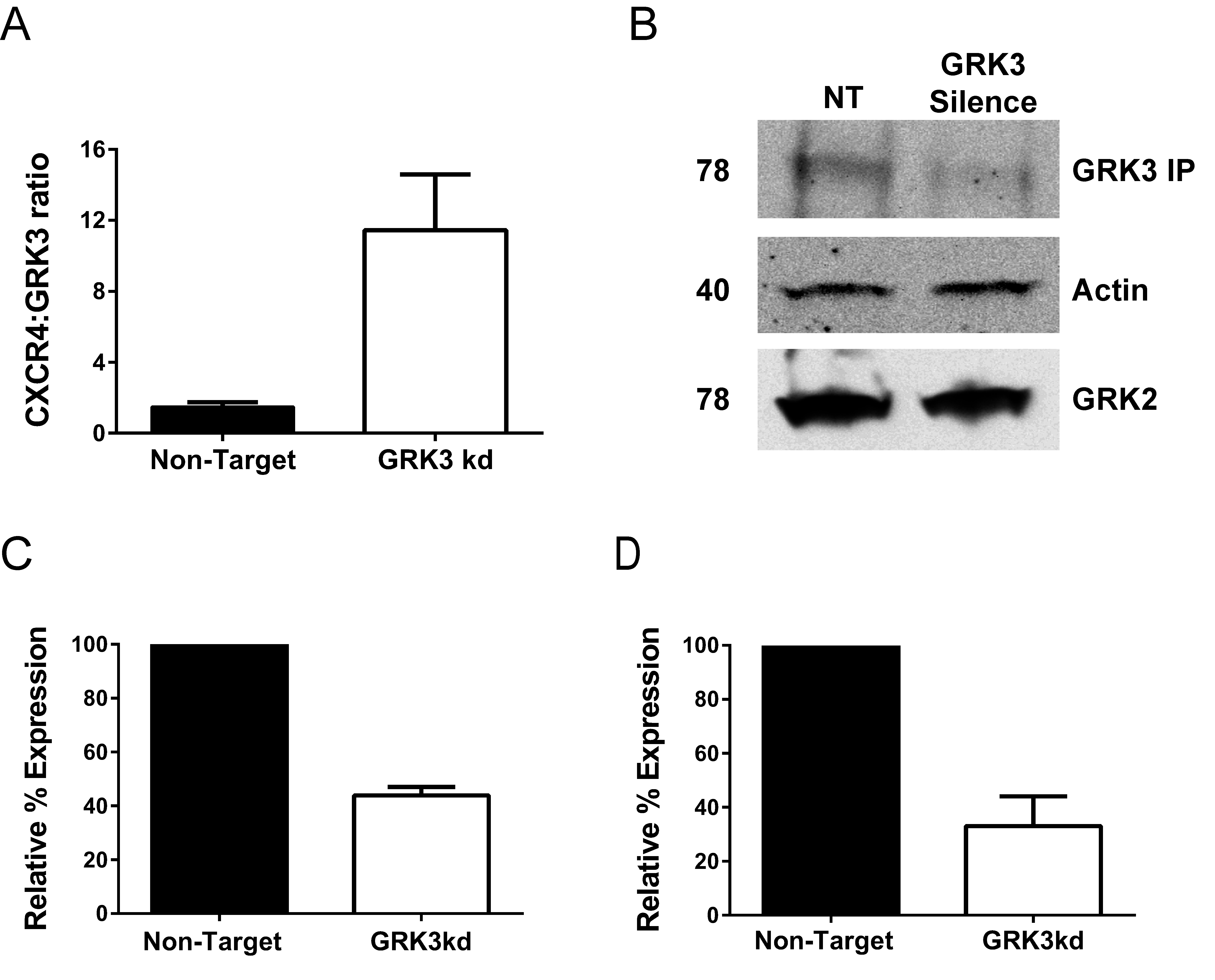

Supplement: S1 Fig — (A) 66cl4-luc murine mammary tumor cells were stably transduced with lentiviral GRK3 shRNA or non-target control plasmids and were analyzed by qRT-PCR to determine the mRNA expression levels of GRK3 and CXCR4 after normalization to the IDUA housekeeping gene. Data is expressed as a ratio of CXCR4 to GRK3. 66cl4-luc Control n = 3, 66cl4-luc GRK3-deficient n = 4. (B) Representative GRK3 Western blot showing shRNA silenced GRK3-deficient 66cl4-luc cells compared to controls after immunoprecipitation. Blots were stripped and reprobed to confirm equal loading. Shown is actin blot of IP supernatant lanes. (C) Prior to implantation into Balb/c mice for in vivo studies, GRK3 is silenced approximately 50–60% in 66cl4-luc cells versus control as determined by qRT-PCR (n = 4). (D) GRK3 gene silencing was validated at 6 week experiment termination by qRT-PCT (n = 4). GRK3 mRNA expression was normalized to IDUA housekeeping gene and relative % expression determined by ΔΔCT method. All error bars represent SEM. (TIF) [file pone.0152856.s001.tif]

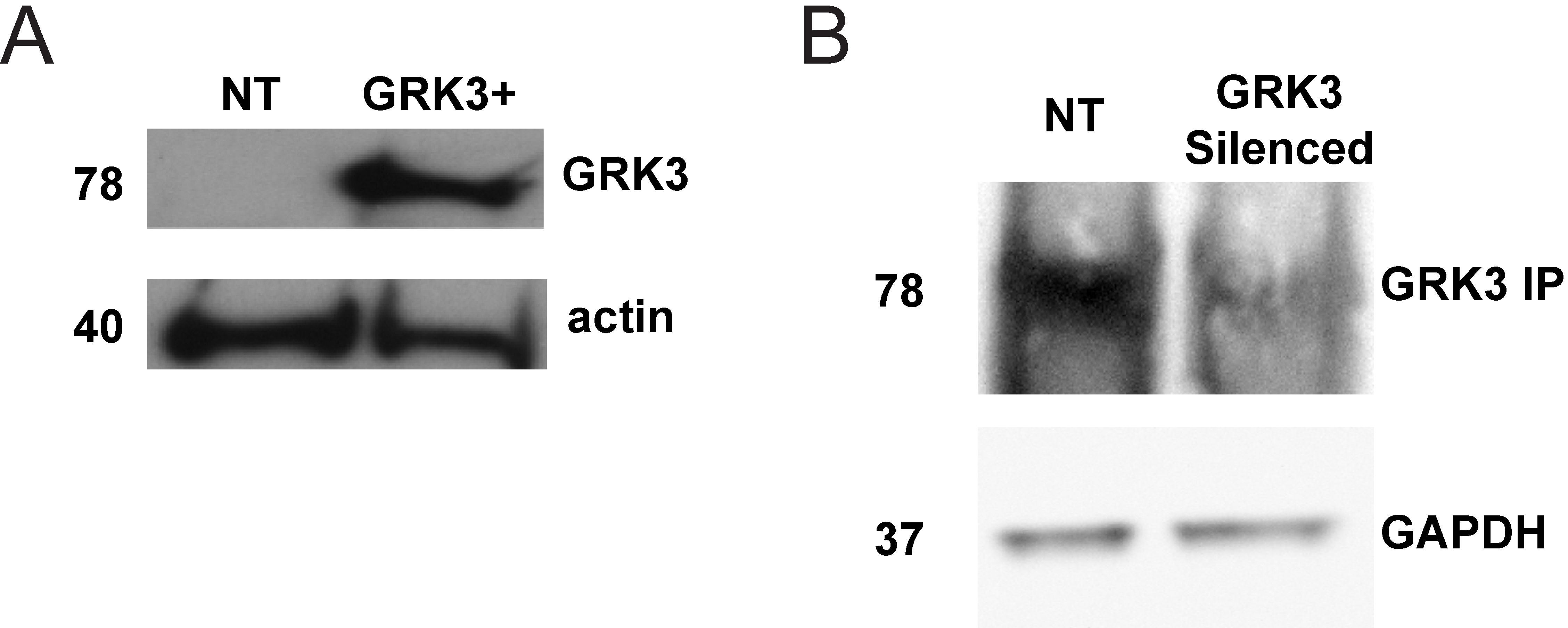

Supplement: S2 Fig — (A) Western blot depicts GRK3 protein overexpression in MDA-MB-231 cells; lysates were also blotted for actin as a loading control. (B) GRK3 Western blot of MDA-MB-468 cell lysates that were stably transduced with lentiviral non-target control plasmid (NT) or GRK3 shRNA (GRK3 silenced). Lysates of equal protein concentration (determined by Protein BCA assay) were immunoprecipitated using anti-human GRK3 monoclonal antibody (Abgent). Immunoprecipitation samples, top, show GRK3 protein knockdown in the shRNA-silenced MDA-MB-468 cells. GADPH in IP supernatants, bottom, confirm equal loading. (TIF) [file pone.0152856.s002.tif]

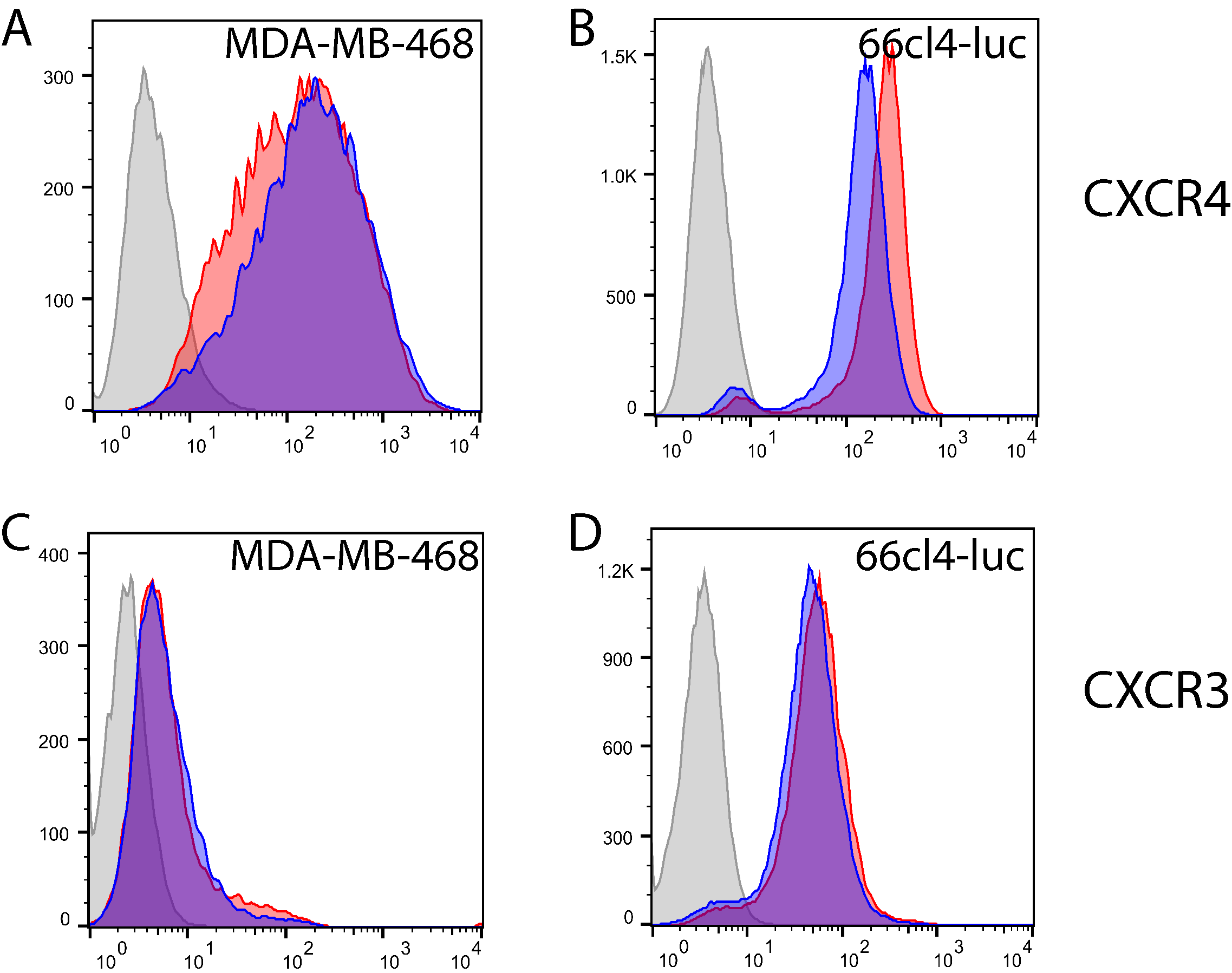

Supplement: S3 Fig — Human breast cancer line MDA-MB-468 (A, C) and murine breast cancer line 66cl4-luc (B, D) that have been stably transduced with GRK3 shRNA (red) or a control sequence (blue) and were stained for CXCR4 and CXCR3 and analyzed by flow cytometry. For human cells, antibodies used were: mouse anti-human CXCR4-PE (clone 12g5, isotype mouse 2a PE, Biolegend), anti-human CXCR3-APC (clone 49801, isotype mouse G1 APC, R&D Systems), and for mouse cells, rat anti-mouse CXCR4-PE (clone 2b11, isotype rat 2b PE, eBioscience) and rat anti-mouse CXCR3-APC (clone 220803, isotype rat 2a, R&D Systems). Negative controls were stained using equivalent amounts of isotype color controls. Shown are representative histograms of 3 independent experiments. (TIF) [file pone.0152856.s003.tif]

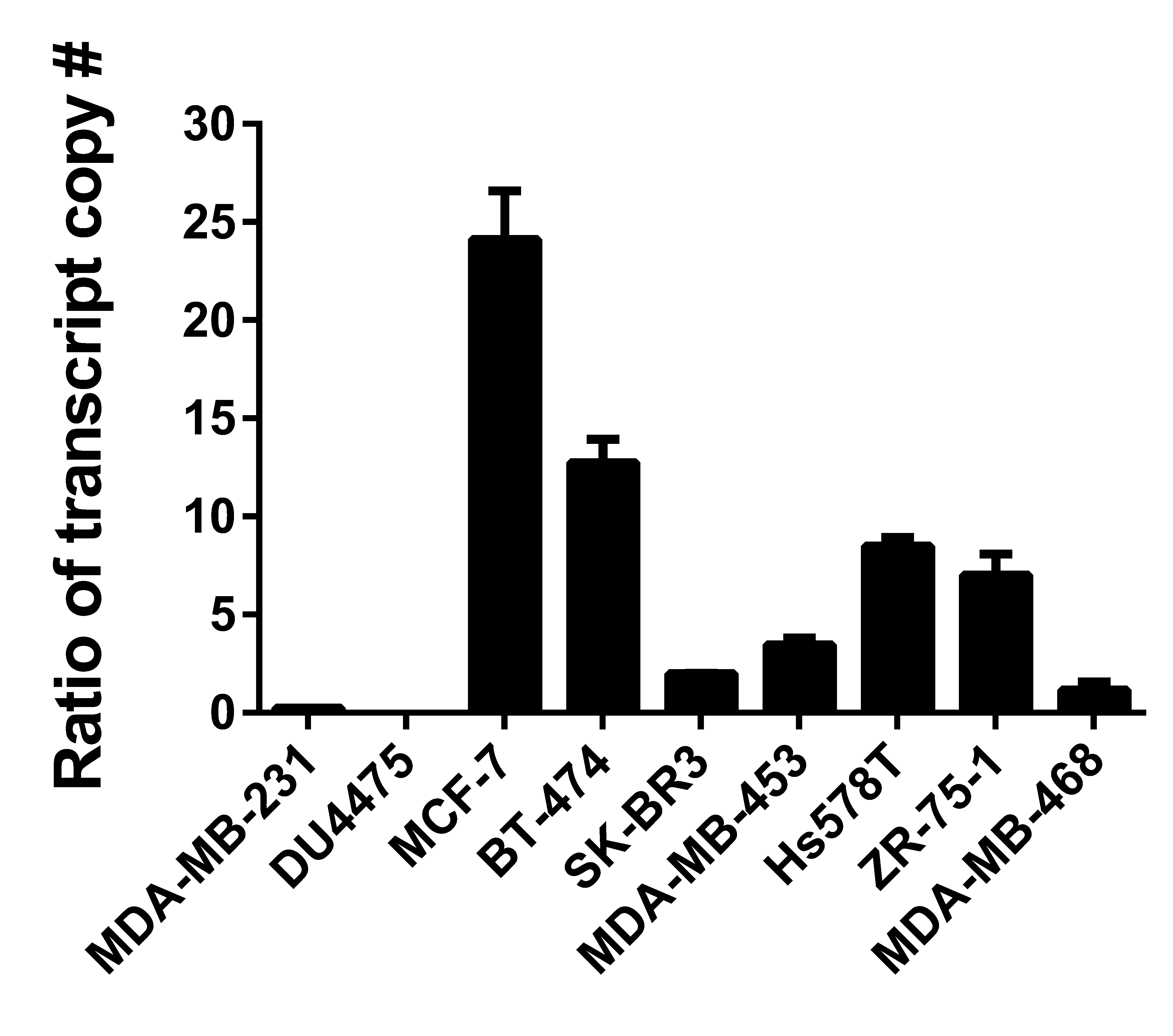

Supplement: S4 Fig — Human breast cancer lines (in descending order moving from left to right of most highly metastatic to least metastatic) were analyzed by quantitative real time PCR to determine the mRNA expression levels of GRK3 and CXCR7. Transcript copy number was determined using the standard curve method. Data shown are the average of two to four independent experiments. Error bars represent the SEM. (TIF) [file pone.0152856.s004.tif]

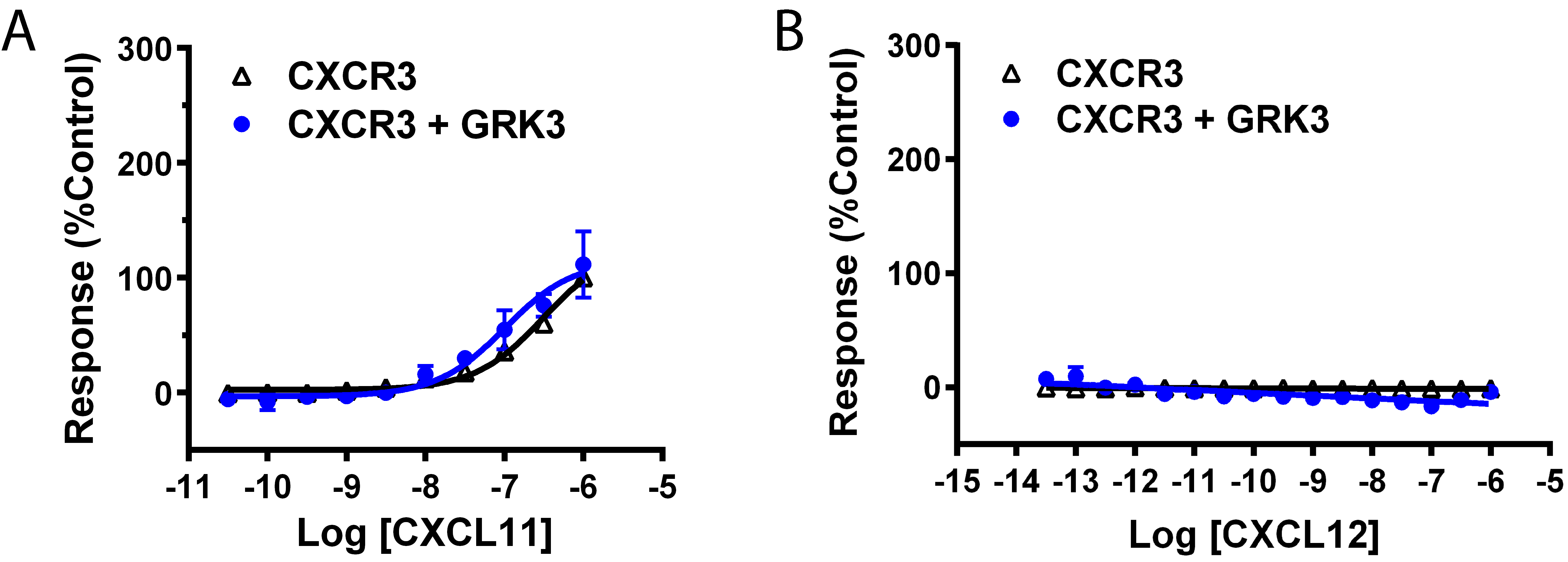

Supplement: S5 Fig — (A) Using a TANGO arrestin-recruitment assay, HTLA cells were transfected with either CXCR3 alone or CXCR3 plus GRK3 as detailed in the Materials and Methods. Cells were plated in a 384 well plate and stimulated with CXCL11 at the indicated Molar concentrations. Luminescence was measured 24 hours post-stimulation. Error bars represent +/- SEM (n = 3). (B) HTLA cells were transfected with either CXCR3 alone or CXCR3 plus GRK3 as in (A). Cells were plated in a 384 well plate and stimulated with CXCL12 at the indicated Molar concentrations. Luminescence was measured 24 hours post-stimulation. Error bars represtent +/- SEM (n = 3). (TIF) [file pone.0152856.s005.tif]

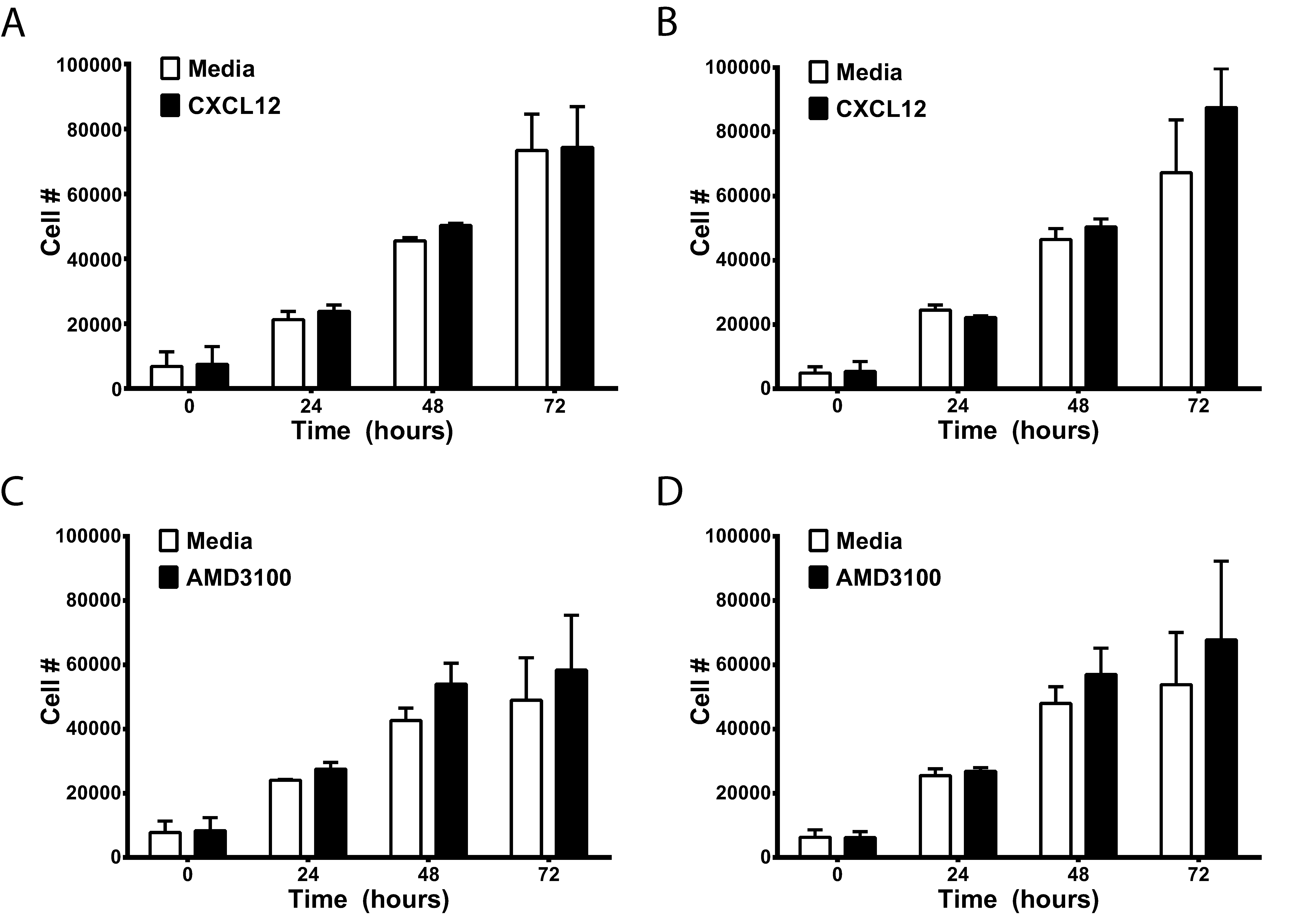

Supplement: S6 Fig — Viable cell density was measured by colormetric assay (Cell Counting Kit-8, Dojindo) at indicated times and cell number estimated by standard curve (n = 2 + SEM). 66cl4-luc non-target control (A) and GRK3-silenced (B) cell proliferation was tested with or without exogenous CXCL12 (100 ng/ml) added to the culture media. Since 66cl4-luc cells make large amounts of endogenous CXCL12 (Fig 6), 66cl4-luc non-target control (C) and GRK3-silenced (D) cell proliferation was also tested in presence or absence of CXCR4 antagonist AMD3100 (5 μg/ml). (TIF) [file pone.0152856.s006.tif]

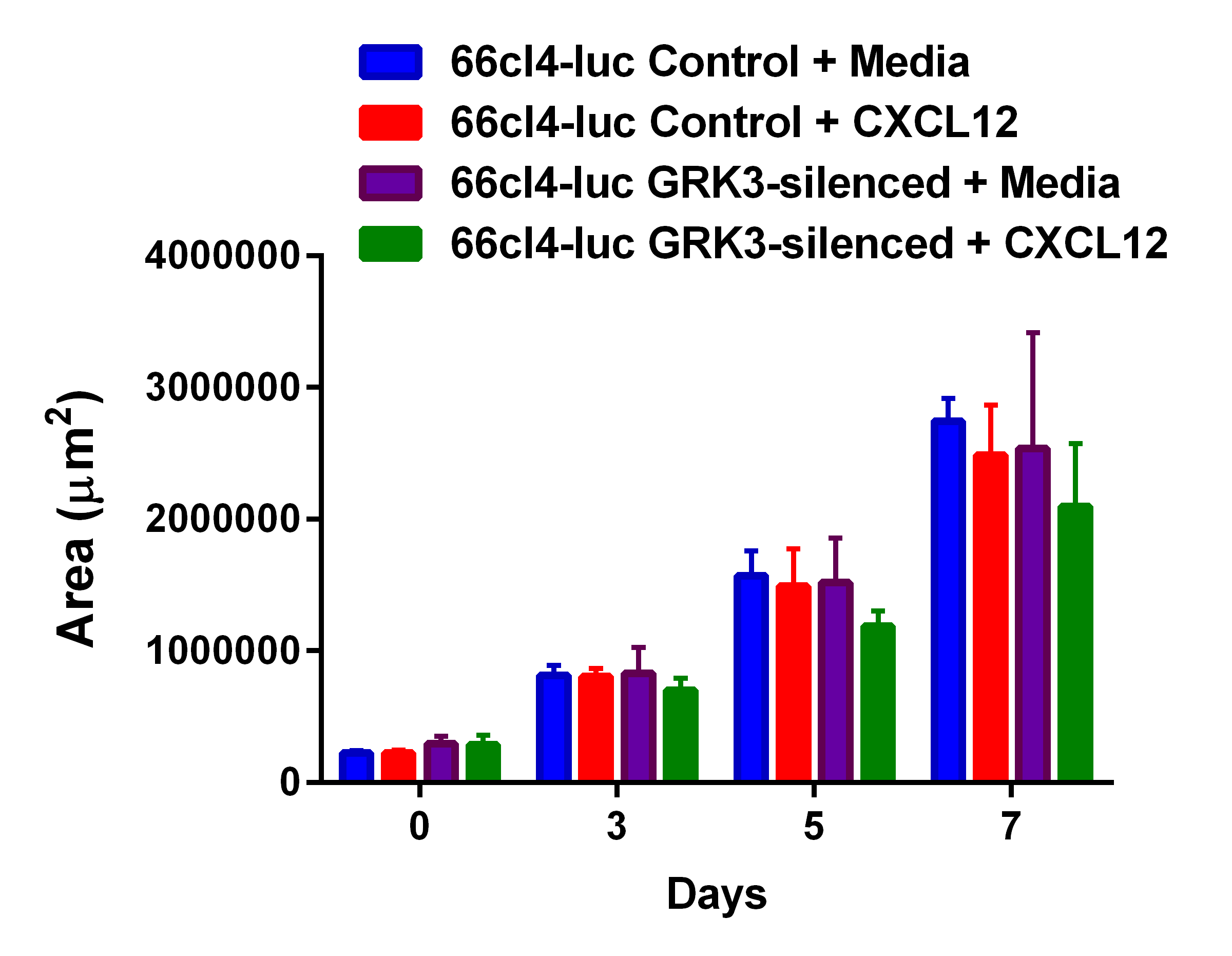

Supplement: S7 Fig — 66cl4-luc murine breast cancer cells were tested using the Cultrex 3D Spheroid Cell Invasion Assay (Trevigen) according to manufacturer’s suggestions. Briefly, cells were allowed to assemble into spheroids for 3 days. Invasion matrix and media (+/- CXCL12) were added and images captured (invasion Day 0). Images were captured at the days indicated and analyzed using Image J software as described in the product insert. Data shown is the mean of two independent experiments (error bars +/- SEM). (TIF) [file pone.0152856.s007.tif]
