# Supplementary material for: G Protein Coupled Receptor Kinase 3 Regulates Breast Cancer Migration, Invasion, and Metastasis
Source: PLoS One. 2016 Apr 6;11(4):e0152856. doi: 10.1371/journal.pone.0152856 (PMC4822790; doi:10.1371/journal.pone.0152856)
Supplement: S1 Methods — (DOCX) [file pone.0152856.s008.docx]

**S1 Methods: Supplemental Information**

**Western blotting**.  Cells were lysed in RIPA buffer supplemented with fresh phosphatase inhibitors: 2 μM Na3VO4, 10 μM NaF, 2 μM Sodium Pyrophosphate, and the Roche complete protease inhibitor cocktail (Roche, IN).  Protein concentration was determined by Pierce BCA Protein Assay Reagent (Thermo Scientific, Waltham, MA).  Proteins were resolved by SDS-PAGE and transferred to Amersham Hybond nitrocellulose membranes.  MDA-MB-231 membranes were blotted using mouse anti-human GRK3 (MAB4785, R&D Systems, Minneapolis, MN).  For cells that expressed low levels of endogenous GRK3 (MDA-MB-468 and 66cl4-luc) immunoprecipitation was necessary.  Briefly, 66cl4-luc cells were lysed as described and protein concentration quantified.  Equal amounts of protein per sample (1.0 mg) were immunoprecipitated overnight with anti-GRK3 (AP7005a, Abgent, San Diego, CA) and 50 μl Protein A/G Plus Agarose (Santa Cruz Biotechnology, CA). Immunoprecipitates and supernatant controls for 66cl4-luc were resolved for Western blot using SDS-PAGE and blotted using anti-GRK3 (MAB4785, R&D).  For loading controls, blots were stripped and reprobed with anti-beta-actin (sc-47778, Santa Cruz) to show equal loading of the IP supernatants.  MDA-MB-468 cells required that GRK2 was pre-cleared from 0.5 mg (total protein) lysates (using Santa Cruz, sc-562).  The pre-cleared MDA-MB-468 lysates were immunoprecipitated overnight with anti-GRK3 (AP7005a, Abgent, San Diego, CA) and 50 μl Protein A/G Plus Agarose (Santa Cruz Biotechnology, CA) and blotted with anti-GRK3 (sc-563, Santa Cruz Biotechnology, CA).   For loading controls, blots were stripped and reprobed with anti-GAPDH (2275-PC, Trevigen, Gaithersburg, MD) to show equal loading of the IP supernatants.

**Quantitative Real Time PCR (qRT-PCR) of 66cl4-luc mammary tumor cells.** Cells were grown in appropriate culture media to 75% confluence. Total RNA was prepared using a Qiagen RNeasy kit and cDNA synthesized with Superscript II reverse transcriptase (Invitrogen) followed by qRT-PCR. Fold differences in expression were calculated using the 2^-ΔCt^ method compared to housekeeping gene IDUA. Transcript copy number was determined using the standard curve method. Mouse primers used included GRK2 forward ATGCATGGCTACATGTCCAA, GRK2 reverse ATCTCCTCCATGGTCAGCAG, GRK3 forward TGCCAGATACCTCTGCATCA, GRK3 reverse TGCTATTGGAGACCTCCTGG, IDUA forward GCATCCAAGTGGGTGAAGTT, IDUA reverse CATTGAGCAGGTCCGGATAC, CXCR4 forward ACTCACACTGATCGGTTCCA, and CXCR4 reverse AGGTGCAGGTAGCAGTGACC. Human primers for qRT- PCR are listed as 5’to 3’ sequence as follows: Human GRK2 forward ACTTCAGCGTGCATCGCAT, GRK2 reverse GCTTTTTGTCCAGGCACTTCAT, GRK3 forward AAGCCTTCGAGGTGACATTTTT, GRK3 reverse GCAACCATAAACTTCCCCGAATC, IDUA forward CTCGGGCCACTTCACTGAC, IDUA reverse CAGTCCGTACCTACCGATGTAT, CXCR7 forward TGCATCTCTTCGACTACTCAGA, CXCR7 reverse GGCATGTTGGGACACATCAC, CXCR4 forward CTCACTGACGTTGGCAAAGA, and CXCR4 reverse AGGAAGCTGTTGGCTGAAAA.

**CXCR4 and CXCR3 Flow Cytometry**

Stable, lentiviral shRNA-transduced breast cancer cell lines MDA-MB-468, and 66cl4-luc were serum-starved and detached using a non-enzymatic method (Versene). Cells were washed once in PBS, then fixed for 15 minutes in 2% paraformaldehyde. Fixed cells were washed three times in PBS then blocked with 1% BSA for 15 minutes. For human cells, antibodies used were: mouse anti-human CXCR4-PE (clone 12g5, isotype mouse 2a PE, Biolegend), anti-human CXCR3-APC (clone 49801, isotype mouse G1 APC, R&D Systems), and for mouse cells, rat anti-mouse CXCR4-PE (clone 2b11, isotype rat 2b PE, eBioscience) and rat anti-mouse CXCR3-APC (clone 220803, isotype rat 2a, R&D Systems). Samples were analyzed using a Beckman Coulter CyAn ADP cytometer.

**ANCOVA Linear Regression Model**

In the statistical analysis of kinetic migration data, the comparison between Control and GRK3 groups was carried out using the difference of the controlled values (CXCL12-media) vs the difference of the GRK values (CXCL12-media). Several model fitting procedures were considered. The simplest is the analysis of covariance model (ANCOVA),([1](#_ENREF_1)) which is a linear regression model involving the response Y (CXCL12-media) regress on TIME (a continuous variable) and GROUP (Control, GRK3: a factor or binary variable). The model is given by

Y = b0 + b1*TIME + b2*GROUP + b3*(TIME*GROUP) + error

The comparison is conducted by testing the following hypotheses:

1. H0: b2=b3=0. If this hypothesis is not rejected (or both coefficients are not significantly different from zero), then there is no GROUP effect and therefore the two do not differ significantly. If H0 is rejected, then the two differ significantly: either b2 or b3 or both are different from zero significantly. This is case for the three data sets. MDA-MB-231 data shows a significant different slope values between control and GRK (b3 is highly significantly different from zero, p<0.001). MDA-MB-468 data have highly significant results for both b2 (p<0.001) and b3 (p<0.001). 66cl4-luc data has a highly significant b2 (p<0.001).

2. H0: b2=0. If this hypothesis is not rejected (or b2 is not significantly different from zero), then the groups share the same baseline value. This is the case for data labeled as MDA-MB-231 (p=0.255).

3. H0: b3=0. If this hypothesis is not rejected (or b3 is not significantly different from zero), then the groups share the same slope or the rate of change value. This is the case for data labeled as 66cl4-luc (p=.872).

To account for the nonlinear TIME effect, a quadratic term TIME*TIME is added to the above model. This only improves the fit slightly (R-squared was up by 3% from 0.41). All the results stated above remain unchanged. A more elaborated approach is to consider a linear mixed model approach for growth-curve or longitudinal analysis. There will be more assumptions involved in the estimating and testing procedures, the method is more complicated to describe and the results will be reported upon request.

References:

1. Kutner MH. Applied linear statistical models. 5th ed. Boston: McGraw-Hill Irwin; 2005. xxviii, 1396 p. p.
